# Genome-wide association studies: assessing trait characteristics in model and crop plants

**DOI:** 10.1007/s00018-021-03868-w

**Published:** 2021-07-01

**Authors:** Saleh Alseekh, Dimitrina Kostova, Mustafa Bulut, Alisdair R. Fernie

**Affiliations:** 1 Max-Planck-Institute of Molecular Plant Physiology, Am Mühlenberg 1, 14476 Potsdam-Golm, Germany; 2 Center of Plant Systems Biology and Biotechnology, 4000 Plovdiv, Bulgaria; 3 Maritsa Vegetable Crops Research Institute (MVCRI), Plovdiv, Bulgaria

**Keywords:** GWAS, Genetic architecture, Quantitative trait loci, Crop species

## Abstract

GWAS involves testing genetic variants across the genomes of many individuals of a population to identify genotype–phenotype association. It was initially developed and has proven highly successful in human disease genetics. In plants genome-wide association studies (GWAS) initially focused on single feature polymorphism and recombination and linkage disequilibrium but has now been embraced by a plethora of different disciplines with several thousand studies being published in model and crop species within the last decade or so. Here we will provide a comprehensive review of these studies providing cases studies on biotic resistance, abiotic tolerance, yield associated traits, and metabolic composition. We also detail current strategies of candidate gene validation as well as the functional study of haplotypes. Furthermore, we provide a critical evaluation of the GWAS strategy and its alternatives as well as future perspectives that are emerging with the emergence of pan-genomic datasets.

**Supplementary Information:**

The online version contains supplementary material available at 10.1007/s00018-021-03868-w.

## Genome-wide association studies (GWAS)

It was reported on 11 January 2019 that for humans 3730 GWAS studies had been published with a total of 37 730 single nucleotide variations and 52 415 unique SNV-trait associations above a genome-wide significance threshold [1, 2]. Analysis of the staggering increase in the number of associations in the time-lapse figure on the GWAS catalog website (https://www.ebi.ac.uk/gwas/) suggests that these numbers have likely increased at least threefold demonstrating the tremendous uptake of this method in recent years. Indeed, as evidenced by the numbers given above since the first GWAS for age-related macular degeneration was published in 2005 [3], well over 50 000 associations of genome-wide significance (*P* < 5 × 10^–8^) have been reported between genetic variants and common diseases and traits [1]. Among these studies risk loci for a vast number of diseases and traits, including anorexia nervosa [4], body mass index [5], cancers and their sub-types [6, 7], coronary diseases [7], inflammatory bowel disease [8], insomnia [9], type 2 diabetes mellitus [10], and schizophrenia [11], have been reported. Indeed, the number of replicable associations is now dramatically higher than those available in the pre-GWAS era [12]. The rapid uptake of GWAS in plants is similar. Indeed, since early studies on flowering time and pathogen resistance [13], single feature polymorphism [14], and recombinant and linkage disequilibrium [15], well over 1000 GWAS studies have now been published in plants [16, 17]. The data from many of these have subsequently been uploaded to the AraGWAS catalog database [18]. In this article we will provide a review of these studies in plants splitting them into four major categories: (1) biotic resistance, (2) abiotic tolerance, (3) yield associated traits, and (4) metabolic composition. We will document strategies of validation and cross-validation and outline how results from these studies are being exploited both as a route by which to gain mechanistic understanding of various biological processes and one to improve agriculture. Finally, we outline alternatives to the GWAS approach as well as providing a prospective for its future application. However, before doing so we feel it highly important to provide a brief overview of the technique itself.

## The GWAS approach

**Fig. 1:**
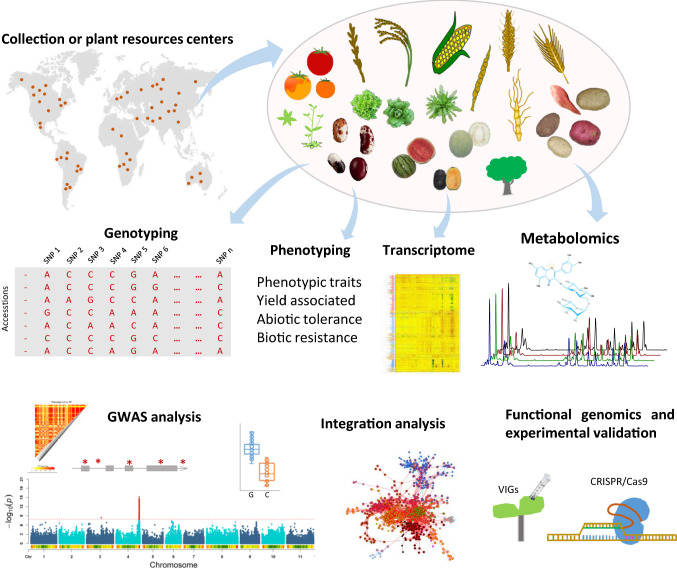
A schematic view of GWAS in plants

The aim of GWAS is exceedingly simple—namely to detect association between allele or genotype frequency and trait status. The first step of such analysis is to identify the traits to be scored and select an appropriate study population considering both the size of the population and the amounts of genetic and trait variance that it possesses (Fig. 1). Depending on whether using a novel population or one that is already well studied genotyping may or may not be necessary. It can be carried out using single nucleotide polymorphism (SNP) arrays combined with imputation [19] or via whole-genome sequencing [2]. Association tests are then used to identify genomic regions that associate with the variance of the phenotype of interest at genome-wide significance with meta-analysis often used to increase the statistical power to detect associations. The first GWAS was performed by Klein et al. [3], who identified a variant of the Complement Factor H gene as being strongly associated with age-related macular degeneration. Within the last 15 years it has been powerful in dissecting the genetic basis for variation in a range of complex phenotypes including disease in humans and animals and physiological and agronomic traits in plants [20–26]. That said population structure and unequal relatedness between individuals can result in spurious associations and thereby false discoveries. To combat this problem considerable effort has been made to statistically account for population structure [27, 28]. For example, in mixed linear models (MLM), population stratification is fitted as a fixed effect, while kinship among individuals is incorporated via the variance–covariance structure of the random effect for the individual [29, 30]. Indeed the MLM method is now firmly established in GWAS since it has proven effective in correcting for the inflation of small genetic effects and controlling bias caused by population structure. Generally such models are carried out with single-locus test, however, multi-locus mixed models have been developed which perform well [31, 32]. While also commonly used single nucleotide polymorphism (SNP)-based GWAS suffers from oft-overlooked interactions between SNPs within a gene and also weak signals aggregating within related SNP sets [33]. To limit such problems, haplotype-based GWAS and gene-based GWAS have been developed which has high statistical power to identify causal haplotypes and demonstrated to be able to identify new candidates for complex traits albeit being less capable of detecting QTL than SNP-based GWAS especially so for rare alleles [34–36]. All these methods are based on the assumption that phenotype and marked effects follow a normal distribution. Two further developments are worthy of note. The Anderson Darling test is a complementary method, which is particularly useful for moderate effect loci or rare variants and with abnormal phenotype distribution [37] while statistics-based fine-mapping strategies have also been developed [38].

Initial excitement surrounding GWA cooled considerably on the appreciation of the above-mentioned facts that GWAS loci often have small effect sizes and explain only a modest proportion of heritability [39]. However, this missing heritability is, at least as long as large and varied populations as used, in fact rather small. What is clear is that the larger the population and the larger the number of SNPs the greater the chance of a successful result with empirical evidence demonstrating that for each complex trait there is a threshold sample size above which the rate of locus discovery accelerates in GWAS [40, 41]. It is important to note, however, that the value of biological insight gained from GWAS is in no way proportional to the strength of association, a fact that provides a strong argument for the value of finding subtle associations in ever larger sample sizes [42]. As stated above genetic variants can be genotyped in many different ways but by far the most predominant are SNP arrays and whole-genome sequencing (see Fig. 1). Given the lowering sequencing costs the latter is beginning to become more frequent. The advantages of SNP arrays, other than their lower costs are the fact that it is highly accurate with a well-established pipeline for analysis. By contrast, although less accurate and more expensive whole-genome sequencing provides coverage also of rare variants and even if the sample size is large enough ultra-rare variants. In addition fine mapping is easier with whole-genome sequencing, however, these advantages come at the cost of higher computational costs including a higher multiple testing burden [2]. To offset some of the limitations of SNP-based GWAS sophisticated tools for genotype imputation have been developed which allow genotypes or untyped variants to be predicted. If the size of the reference panel is large enough and a subset is well sequenced this imputation has been demonstrated to be highly reliable [43, 44]. Given this fact it is not surprising that both approaches currently retain utility. However, whole-genome sequencing is the gold standard in GWAS [45–47] and has the potential to resolve many of the limitations of the method (for example the identification of missed signals, accounting for population stratification, identification of ultra-rare mutations as well as gene–gene and gene-environment interactions and to explain even more of the missing heritability). We will discuss this in detail when we compare GWAS with other strategies to link genotype with phenotype in *Limitations of GWAS an alternative approaches to GWAS* below. Having provided a general introduction to the approach above we will, use early case studies in Arabidopsis that span a wide range of phenotypic traits to illustrate it in detail below before providing a more comprehensive overview of its use in other species.

## Early studies of GWAS in Arabidopsis

As for many studies in the last 40 years the initial applications of GWAS in plants were in Arabidopsis. The very earliest studies focused on single feature polymorphism [14] and recombination and linkage disequilibrium [15], but a far more diverse range of phenotypes have been studied in the interim. The study of Borevitz et al. used hybrization to a microrarray as a means to assess genomic DAN diversity of 23 ecotypes in comparison to the reference ecotype Col0 allowing assessment of over 77 000 single feature polymorphisms [14]. Similarly, that of Kim et al. analyzed linkage disequilibrium in a sample of 19 Arabidopsis accessions using approximately 350 000 non-singleton SNPs demonstrating the presence of clear recombination hotspots in intergenic regions [15]. Currently, in Arabidopsis results of > 400 GWAS covering an exhausting range of phenotypes are curated in the AraGWAS catalog [18]. To highlight a few recent studies we will focus on growth, metabolism, defense, and evolution of tolerance to abiotic stress [48–52]. Growth and metabolism have been evaluated in association with enzyme activities of primary metabolism [48], while primary [51] and secondary metabolite contents [49, 50] have also been studied via the use of metabolomics approaches. All of these studies have provided greater insight into the interplay between metabolism and growth on one hand and defense on the other [53], with both difference in the levels of defense metabolites and altered alleles of ACCELERATED CELL DEATH6 suggesting a trade-off between metabolism and defense. Abiotic stress has also been much studied in Arabidopsis populations with the recent tour-de-force work of Exposito-Alonso representing a beautiful example of the power of this approach [52]. These authors evaluated 517 Arabidopsis ecotypes grown in Spain and Germany simulating high and low precipitation at each site quantifying survival and fecundity and thereafter performing a GWAS in the quantified selection coefficients. They observed that a significant proportion of the climate-driven natural selection was predictable form signatures of local adaptation since genetic variants were found in geographical areas with climates more similar to the experimental sites were positively selected. These data thus allowed them to forecast that with the increased frequency of drought and temperature in Europe such positive selection will sweep Northwards across Europe.

While the above studies represent impressive proof-of-concept studies and additionally greatly refined our understanding of the genotype-to-phenotype interface [16], as we will detail in the following sections it has been adopted in cereal crops (rice [22, 54] maize [55, 56], wheat [57] and barley [58]) as well as soybean [59–61], cotton [62, 63], tomato [25, 26], cucumber [64, 65], sesame [66], peanut [67], peach [68], melon [69], tea [70], and lettuce [71, 72]. As we will elaborate in the next four sections, these studies, alongside the purpose-developed populations, catalogs of allelic variants, and corresponding genotype–phenotype associations, provide unprecedented resources for understanding crop functional genomics [33].

## Adoption of GWAS in crop species (i) biotic resistance

 In the above section we have detailed some studies evaluating biotic stress in Arabidopsis. In crops this is of massive importance with 20–40% yield losses predicted to be caused by biotic interactions annually. While considerable success has been made by breeding efforts—notably the introgression of wild species alleles conferring resistance [73, 74]. Critically the collection of broad populations for, among others, the species listed above renders GWAS, an attractive approach for the identification of further genes of interest for this purpose. As can be seen in Supplementary Table 1, there are already a vast number of such studies covering many species. Here, we will highlight only the few summarized in Table 1.

**Table 1:** List of selected genome-wide association studies in Arabidopsis and major crop plants

	Species (common name)	Panel size [markers]	Trait [associations]	References	Validation
Arabidopsis	*A. thaliana* (Arabidopsis)	96 [200,000]	Metabolites [**]	[148]	−
	*A. thaliana* (Arabidopsis)	314 [199,455]	Primary metabolites [117]	[51]	+
	*A. thaliana* (Arabidopsis)	91 [4,000,000]	Drought [**]	[149]	−
	*A. thaliana* (Arabidopsis)	349 [214,051]	Central metabolism and plant growth [131]	[48]	+
	*A. thaliana* (Arabidopsis)	309 [199,455]	Darkness [123*]	[49]	+
	*A. thaliana* (Arabidopsis)	517 [1,353,386]	Environmental adaptation [6,660]	[52]	+
Metabolite QTL	*Z. mays* (Maize)	513 [56,110]	Specialized metabolites [16]	[150]	+
	*Z. mays* (Maize)	368 [1,030,000]	Metabolites [74*]	[55]	+
	*Z. mays* (Maize)	368 [560,000]	Metabolites [882*]	[151]	-
	*Z. mays* (Maize)	282 [29,000,000]	Specialized metabolites [**]	[103]	-
	*Z. mays* (Maize)	368 [560,000]	Lipid biosynthesis [139]	[106]	-
	*O. sativa* (Rice)	529 [6,400,000]	Metabolites [634]	[152]	+
	*O. sativa* (Rice)	502 [3,900,000]	Metabolites [105]	[20]	+
	*Solanum *spp (Tomato)	398 [2,014,488]	Flavor [251]	[25]	+
	*H. vulgare *L*. *var.* nudum* (Tibetian Hulles Barley)	196 [19,248,055]	Metabolites [90*]	[58]	+
	*L. sativa* (Lettuce)	189 [16,611]	Primary metabolites [154*]	[153]	+
Yield associated	*G. hirsutum* (Cotton)	258 [1,871,401]	Yield-related traits [119*]	[62]	−
	*G. max* (Soybean)	809 [10,415,168]	Agronomic traits [245*]	[89]	−
	*L. batatas* (Sweet potato)	358 [33,068]	Root-related traits [34]	[91]	−
	*O. sativa* (Rice)	242 [700,000]	Agronomic traits [10*]	[88]	−
	*P. vulgaris* (Common bean)	683 [4,811,097]	Yield associated traits [505*]	[154]	−
Biotic stress	*Z. mays* (Maize])	5,000 [1,600,000]	Resistance to Southern Leaf Blight [245*]	[56]	−
	*Z. mays* (Maize)	318 [542,438]	*Rhizoctonia solani* resistance [28]	[75]	+
	*G. max* (Soybean)	330 [25,179]	*Sclerotinia sclerotiorum* resistance [38]	[155]	−
	*O. sativa* (Rice)	67 [2,576]	Blast resistance [36]	[156]	+
	*T. aestivum* (Wheat)	2,300 [49,905]	Rust resistance [161/33]	[77]	−
	*T. turgidum *ssp*, Dicoccum* (Emmer Wheat)	176 [5106]	*Puccinia striiformis* resistance [51*]	[76]	−
Abiotic stress	*O. sativa* (Rice)	553 [304,877]	Salinity tolerance [**]	[82]	−
	*O. sativa* (Rice)	68 [27,192]	Flooding tolerance [6*]	[157]	+
	*O. sativa* (Rice)	1,033 [289,231]	Cold tolerance [5*]	[85]	+
	*O. sativa* (Rice)	117 [1,531,224]	NUE-related agronomic traits [7]	[83]	+
	*Z. mays* (Maize)	338 [56,110]	Metabolites under low Pi [178]	[84]	+

Expanded list is provided in Supplementary Table 1

* Number of QTLs, ** several associations,  +  experimental validation of the genes/s, − no experimental validation of the candidate genes or loci

Starting with studies in our major cereals we will describe two studies each for maize and wheat and one for rice before highlighting the possible value of this approach in two less studied crops. The first study in maize used the nested association mapping population to identify 32 QTL with small additive effects on southern leaf blight with many being within or near genes previously shown to be involved in plant disease resistance [56]. More recently, GWAS revealed that the F-Box protein ZmFBL41 which interacts with ZmCAD encoding the terminal enzyme of the monolignol pathway which if active restricts lesion expansion [75]. Similarly, in a GWAS-based study in rice Li et al. found a natural allele of a C_2_-H_2_ type transcription factor that confers broad spectrum resistance. Haplotype analysis (which we will return to it below), revealed that this allele exists in 10% of accessions of rice. This allelic variance was associated to an inhibition of H_2_O_2_ degradation which the authors postulate is responsible for the observed resistance. In Emmer wheat stripe resistance loci that were associated with field resistance in multiple environments with more than half of these representing novel candidate genes that were not found in linkage mapping studies [76]. Meanwhile, a recent large-scale study in 2 300 bread wheat accessions was used to investigate leaf-, stem-, and stripe-rust diseases with both single- and multi-trait GWAS being applied [77]. Importantly, both studies revealed the utility of small effect QTL in achievement of durable resistance.

Of the less studied species, we would highlight two cassava which is actually the fourth largest crop in terms of production globally [78] and pigeonpea an important smallholder crop in India and Africa [79]. For cassava GWAS for cassava mosaic disease and cassava green mite severity were carried out identifying several novel and previously reported associations. For pigeonpea a pangenome was recently published based on 89 accessions and this will surely be a fantastic resource for future studies. Indeed, since so many natural populations are now established it would seem likely that their use as well as those of biparental and multi-parental populations will likely unlock resistance in a wide range of plant-pest combinations and as such will result in the achievement of durable resistance.

## Adoption of GWAS in crop species (ii) abiotic tolerance

Similarly to the above studies aiming to generate more resistant plants considerable research and breeding efforts have been expended on identifying and utilizing allelic variance that confers tolerance to abiotic stresses. As can be seen in Supplementary Table 1, there are already a vast number of such studies covering many species. Here, we will highlight only the few summarized in Table 1 focusing on water and salt stress as well as macronutrient and temperature stress. Arguably, the most important of these is drought stress with yield losses of > 50% being estimated to be due to this stress annually [80]. While water deficiency can devastate crop yields the opposite, i.e., flooding can have the same consequences. The development of varieties of rice that are tolerant of flooding is thus highly desirable. The identification of haplotypes of the SEMIDWARF1 gene that facilitate this [81] presents an excellent example of the power of haplotype analysis following GWAS studies (an analysis type we will return to it below). Similarly in rice, salt stress has been much researched. Al-Tamanini et al. combined high throughput phenotyping of plant growth and transpiration with high-density genotyping if indica and aus diversity panels containing a total of 553 accessions [82]. This study identified a previously undetected loci for salt stress localizing to chromosome 11, thus, providing new insight into early responses to rice salinity and providing hints as to how breeding could alleviate this problem.

Given that nitrogen fertilizer is often over applied to fields often with catastrophic ecological consequences. There is, thus, a pressing need to develop crops exhibiting high nitrogen use efficiency to reduce fertilizer to move towards a more sustainable agriculture. Tang et al. recently identified the nitrate transporter OsNPF6.1 (HapB) as conferring high nitrogen use efficiency in a GWAS experiment conducted on a rice diversity panel [83] with haplotype analysis identifying that this allele had been lost in over 90% of rice varieties. In a similar vein GWAS was used to investigate phosphate use efficiency in maize [84] with metabolomics being utilized in this study to understand how metabolism is reprogrammed under phosphate limitation. The combined work identified phosphoglucose isomerase activity to be a key determinant of phosphate use efficiency suggesting it to be a strong lead gene for lessening the need of P fertilization [84].

Extreme temperatures also often provoke deleterious effects on crop yield. For this reason, GWAS was recently applied to identify genes underlying cold tolerance in a large 1033 accession rice diversity panel [85]. This study resulted in the identification of five cold tolerance related genetic loci with one loci LOC_Os10g34840 being deemed responsible for cold tolerance at the seedling stage with the cold tolerant allele being present in 80% of temperate japonica accessions but only 3.8% of the indica accessions. By contrast, for high temperature tolerance, GWAS discovered genetic factors associated with four production traits in both heat and drought stress environments in common bean (*Phaseolus vulgaris *L.) [86].

## Adoption of GWAS in crop species (iii) yield associated traits

Having addressed the use of association mapping in resistance and tolerance of plants to biotic and abiotic factors, respectively, above it is important to note that considerable research effort has additionally been placed on elucidating the genetic basis of yield associated traits. As for the above traits we have listed several GWAS studies reporting yield associated traits in Table 1 and provide a more extensive list in Supplementary Table 1. An early study tested almost 5000 lines from the maize NAM population described above to identify numerous small effect QTL with a simple additive model being able to predict flowering time [87]. In addition to flowering time, in rice panicle architecture is a key target of selection. A total of 49 panicle phenotypes were recently assessed in 242 tropical rice accessions allowing the identification of ten GWAS peaks but also demonstrating subtle links between panicle size and yield performance [88]. The complexity of agronomic yield was similarly underlined by a study of 84 agronomic traits in a panel of 809 soybean accessions with many of the loci exhibiting complex pleiotropic effects [89]. In upland cotton a GWAS identified two ethylene pathway related genes as associated with increased lint yield with an analysis of population frequencies revealing that the majority of the elite alleles detected were transferred from a mere three founder landraces [62]. Such analyses are not restricted to cereals with analysis even being carried out in long lived species such as Populus trees [90], as well as sweet potato [91] and GWAS confirming the Lin5 association with agronomic yield in tomato [25] that had previously been identified by linkage mapping [92]. It is perhaps not unexpected that the QTL for yield associated traits seem generally not to be conserved across species.

## Adoption of GWAS in crop species (iv) metabolic composition

Combining the developments in sequencing with those in mass-spectrometry-based analytical systems, has rendered understanding of the genetic architecture of metabolism far easier than it was previously [33, 93–95]. Indeed the immense metabolic diversity of plants has made the ideal models for dissecting the genetic bases underlying the regulation of the metabolome with studies progressing from analysis of mutant libraries [96, 97], and the analysis of gene families [98, 99] via the comparison of sister species [100] and species series within taxa [101] to linkage mapping, and association mapping based on next-generation sequencing have been applied to metabolomics studies [33]. By contrast to the QTL for agricultural performance described above, genetic variants controlling natural variation in metabolite accumulation are easier to identify due to both the tremendous diversity apparent across experimental populations [20, 102–105] and the high accuracy of evaluation of metabolite content [95]. As mentioned above a wide range of examples are now published both in cereal and non-cereal crops (Table 1 and Supplementary Table 1). Due to space limitations we limit our discussion to ten of these examples. In maize, GWAS was used to quantify metabolite contents of nearly 1000 mass features in over 700 lines and further allowed the association of metabolite features with kernal size [55] while a more recent study identified four times as many features paying particular attention to the benzoxazinoids and hydroxycitric acids [103]. Earlier a ground-breaking highly comprehensive study on maize kernel oil identified 74 associated loci of which 26 were found that could explain up to 83% of the phenotypic variation using a simple additive mode.

Maize kernel oil is a valuable source of nutrition. In a seminal study, Li et al. examined the genetic architecture of oil accumulation in maize by GWAS using 368 maize inbred lines characterized to contain in excess of 1 million SNPs. In the process, they identified 74 loci associated with kernel oil levels and fatty acid composition. They validated more than half of these in a linkage mapping population and 26 of the conserved loci were annotated as enzymes of oil biosynthesis and could explain up to 83% of the phenotypic variation in this trait [106]. Similarly in rice, secondary metabolism data of 175 accessions identified 323 associations among 143 SNPs and 89 metabolites. While a comparative analysis between maize and rice demonstrated a considerable amount of shared loci associated with metabolites common to both species [20], but of course could not provide information with regard to species-specific metabolites or for that matter genes [33]. The use of this approach in wheat and barley has allowed the definition of the flavonoid biosynthesis pathway in the former and a novel metabolite, thereof, that confers UV-tolerance in hulless barley, respectively. In tomato, GWAS was used in concert with metabolite profiling and taste panels to characterize the genetic architecture of tomato fruit taste [25] and with metabolic and transcript profiling to characterize the changes in the metabolome that occurred during the domestication and improvement processes [26] while a combination of GWAS, a multi-parental breeding population and transgenic lines was used to characterize the control of vitamin E levels in this fruit [107]. To summarize, metabolic GWAS has proven highly informative not only as a means of identify lead genes for engineering of specific metabolite contents but also in beginning to define the biological function of specific metabolites [95]. However, in certain species such as citrus the use of GWAS is not yet tractable most likely due to population structure issues (unpublished), and this fact is important to keep in mind before carrying out labor-intensive studies, on a new species—irrespective of the phenotype studied.

## Validation of candidate gene function

Despite the strong theoretical foundation we discuss above and considerable efforts being taken to address population structure and employ strict probability cut-offs, false-positive associations will still occur due to the enormous number of statistical inferences and other factors which are not taken into account by the simplicity of the approach [17, 108, 109]. As a consequence independent biological validation is required, however, often not provided [17]. That said two forms of validation have been employed in several instances (i) the validation of associations in independent populations or (ii) validation by targeted viral-induced gene silencing, transgenesis and gene editing experimentation. Cross-population validation is currently largely achieved by integrating association mapping in diverse panels or linkage mapping in RIL population(s) or F2 populations. For example, in the recent cloning of ZmCCT9, a QTL which affects maize flowering time [110], the locus was simultaneously identified by NAM [87] and maize-teosinte RIL populations under association and linkage mapping. Moreover, the causal allele—an InDel of a harbinger-like transposon—has also been identified in a 513 line association panel [111] a fact that was cross-validated in the two populations used to map the locus. In a similar example, rice chlorophyll content was mapped in a panel of 529 individuals followed by three customized F2 populations [112]. Other such examples are the metabolomes of maize [113] and in independent studies the QTL underlying total soluble solid content [92, 113] and alterations in the metabolome [26, 93] in tomato and the exquisitely controlled study mentioned above which used GWAS, multi-parental breeding populations and transgenics to confirm QTL for tocopherol contents [107]. The increasing availability of populations which have been characterized should massively increase or capacities to do such experiments which will undoubtedly massively boost our confidence in the results of association mapping studies. In this vein, it is important also to note also the value of cross-species analysis which has already been implemented in cereals [20, 114, 115] and would probably prove tractable in other agronomically important families such as the Brasicacae, Solanaceae, and legumes. Rather than employing the cross-validation approach which can prove incredibly time and labor intensive several other more direct approaches have been taken. For example, the confirmation of many metabolic QTL has been provided by the reduction of the expression of candidate genes via virus-induced gene silencing [93, 95, 116] or alternatively via their transient or inducible expression [20]. Given that the repertoire of species amenable to both methods are currently being considerably expanded. While these are great for select candidates the promise of clustered regularly interspaced short palindromic repeats (CRISPR)/CRISPR associated protein 9 (Cas9) mutant libraries such as those set up for rice [117, 118] and more recently maize [119] should greatly accelerate the functional confirmation of causality. Like the VIGS and transient expression methods, the range of plant species for which multiple publications on the use of CRISPR has seen a steep increase in recent years [119, 120].

## Limitations of GWAS an alternative approaches to GWAS

Despite the great success of the method as evidenced by the wealth of information described above (and in the Supplementary Table 1), GWAS currently has clear limitations the major of which being issues concerning population structure and low-frequency causal alleles leading to false negative results [121]. For example, given that flowering time is a typical adaptive trait and is always confounded (i.e., highly correlated) with population structure, only one gene (ZmCCT) was revealed for flowering time using a diverse association mapping panel consisting of 500 inbred lines [122]. It is widely accepted that many false negatives occur for such confounded traits when correcting for population structure in GWAS [17, 123]. Another example is the demonstration that only five inbred lines in a population of 527 (< 1%) possess functionally alternative alleles at the Brachytic2 locus for plant height [124] rendering it impossible to identify this locus using routine association mapping analysis. Similarly in rice, causal alleles within most of the cloned yield related quantitative trait loci (QTLs) are at low frequency in diverse germplasms (1% for GS3, [125]; 2% for Ghd7, [126–128]; 2% for qGL3, [129]; 6% for TGW6, [130]). Two routes to tackle these issues have been suggested either the development of novel statistical methods for the exploration of rare functional alleles [131–133] or alternatively employing artificially designed populations to balance allelic frequencies and thereby control population structure [87, 134–136]. Given that these have been reviewed in depth recently [17, 137–139]we will not discuss them in detail here.

In addition to the above issues, sometimes non-causative loci show more significant associations in GWAS than the causative ones meaning the causative genes may be distant from the GWAS peaks. Such an occurrence has been reported in a number of plant studies including studies in Arabidopsis [140, 141], sorghum [142], and tomato [143]. Such misleading associations are sometimes known as synthetic associations and are presumed to be caused by linkage drag caused by linkage disequilibrium between common tagged markers and rare causative variants [17, 144]. This may in turn explain the so-called missing heritability issue of GWAS. That said some causes do not follow the rare-allele assumption but trait variation rather appears to be caused by multiple alleles within one gene [34, 142]. Given that mutation constantly generates new variants, multiple independent alleles within one gene leading to the same phenotype could be common. As we state above haplotype- or gene-based methodologies, therefore, have high potential for identifying such situations. That said current haplotype-based association mapping remains imperfect [145] and, moreover, is particularly challenging in plants [17]. Thus improving haplotype analyses will likely prove highly beneficial both at the understanding of the underlying genetics as well as its functional physiological consequence.

## Current and future perspectives for GWAS

The power of genome-wide association studies have successfully identified enormous number of loci associated with phenotypic, expression, and metabolic traits in multiple species. Although, the genetic factors underling some of these associations have been characterized. The vast majority are remain unexplained. The development of next-generation sequencing and bioinformatics tools greatly improved and currently implemented to decipher the genetic diversity of targeted traits. This recently supported by multi‐omics data analysis to enhancing our understanding of phenotypic diversity and its corresponding genetic basis. Combined analyses of phenotypic and transcriptomic data have been utilized to dissect the genetic basses of various metabolic and phenotypic traits see [146]. Moreover, the developments of molecular biology techniques (e.g., CRISPR/Cas9, over‐expression, or genetic complementation) have greatly accelerated the biological functions of the causative genes behind the GWAS hits. Currently, the cross-validation by combing association and linkage (F2, RILs) mapping has already been implemented in crop [25, 147]. Finally, despite molecular and genetic validations are the reliable ways to validate the GWAS results, there are still accompanying challenges need to take into consideration, such as; epistasis, heterosis and environmental factors. Once such factors are assembled, it will improve our chance of understanding the genetic regulation of complex traits, and provide viable targets for crop improvement and breeding.

### Supplementary Information

Below is the link to the electronic supplementary material.

Supplementary file1 (DOCX 636 KB)

## Data Availability

Data associated with a paper are available in the manuscript.

## References

[1] Buniello A (2019). The NHGRI-EBI GWAS Catalog of published genome-wide association studies, targeted arrays and summary statistics 2019. Nucleic Acids Res.

[2] Tam V (2019). Benefits and limitations of genome-wide association studies. Nat Rev Genet.

[3] Klein RJ (2005). Complement factor H polymorphism in age-related macular degeneration. Science.

[4] Duncan L (2017). Significant locus and metabolic genetic correlations revealed in genome-wide association study of anorexia nervosa. Am J Psychiatry.

[5] Yengo L (2018). Meta-analysis of genome-wide association studies for height and body mass index in ∼700000 individuals of European ancestry. Hum Mol Genet.

[6] Milne RL (2017). Identification of ten variants associated with risk of estrogen-receptor-negative breast cancer. Nat Genet.

[7] Sud A, Kinnersley B, Houlston RS (2017). Genome-wide association studies of cancer: current insights and future perspectives. Nat Rev Cancer.

[8] de Lange KM (2017). Genome-wide association study implicates immune activation of multiple integrin genes in inflammatory bowel disease. Nat Genet.

[9] Jansen PR (2019). Genome-wide analysis of insomnia in 1,331,010 individuals identifies new risk loci and functional pathways. Nat Genet.

[10] Suzuki K (2019). Identification of 28 new susceptibility loci for type 2 diabetes in the Japanese population. Nat Genet.

[11] Li Z (2017). Genome-wide association analysis identifies 30 new susceptibility loci for schizophrenia. Nat Genet.

[12] Lohmueller KE (2003). Meta-analysis of genetic association studies supports a contribution of common variants to susceptibility to common disease. Nat Genet.

[13] Aranzana MJ (2005). Genome-wide association mapping in Arabidopsis identifies previously known flowering time and pathogen resistance genes. PLoS Genet.

[14] Borevitz JO (2007). Genome-wide patterns of single-feature polymorphism in *Arabidopsis thaliana*. Proc Natl Acad Sci USA.

[15] Kim S (2007). Recombination and linkage disequilibrium in *Arabidopsis thaliana*. Nat Genet.

[16] Fernie AR, Gutierrez-Marcos J (2019). From genome to phenome: genome-wide association studies and other approaches that bridge the genotype to phenotype gap. Plant J.

[17] Liu HJ, Yan J (2019). Crop genome-wide association study: a harvest of biological relevance. Plant J.

[18] Togninalli M (2020). AraPheno and the AraGWAS Catalog 2020: a major database update including RNA-Seq and knockout mutation data for *Arabidopsis thaliana*. Nucleic Acids Res.

[19] Johnson EO (2013). Imputation across genotyping arrays for genome-wide association studies: assessment of bias and a correction strategy. Hum Genet.

[20] Chen W (2016). Comparative and parallel genome-wide association studies for metabolic and agronomic traits in cereals. Nat Commun.

[21] Horton MW (2012). Genome-wide patterns of genetic variation in worldwide *Arabidopsis thaliana* accessions from the RegMap panel. Nat Genet.

[22] Huang X (2010). Genome-wide association studies of 14 agronomic traits in rice landraces. Nat Genet.

[23] Tian D (2020). GWAS Atlas: a curated resource of genome-wide variant-trait associations in plants and animals. Nucleic Acids Res.

[24] Tian F (2011). Genome-wide association study of leaf architecture in the maize nested association mapping population. Nat Genet.

[25] Tieman D (2017). A chemical genetic roadmap to improved tomato flavor. Science.

[26] Zhu GT (2018). Rewiring of the fruit metabolome in tomato breeding. Cell.

[27] Devlin B, Roeder K (1999). Genomic control for association studies. Biometrics.

[28] Liu X (2016). Iterative usage of fixed and random effect models for powerful and efficient genome-wide association studies. PLoS Genet.

[29] Yu JM (2006). A unified mixed-model method for association mapping that accounts for multiple levels of relatedness. Nat Genet.

[30] Zhao KY (2007). An Arabidopsis example of association mapping in structured samples. PLoS Genet.

[31] Segura V (2012). An efficient multi-locus mixed-model approach for genome-wide association studies in structured populations. Nat Genet.

[32] Wen YJ (2017). Methodological implementation of mixed linear models in multi-locus genome-wide association studies (bbw145, 2016). Brief Bioinform.

[33] Fang C, Luo J (2019). Metabolic GWAS-based dissection of genetic bases underlying the diversity of plant metabolism. Plant J.

[34] Yano K (2016). Genome-wide association study using whole-genome sequencing rapidly identifies new genes influencing agronomic traits in rice. Nat Genet.

[35] Zhang, W.C., et al., *PEPIS: A Pipeline for Estimating Epistatic Effects in Quantitative Trait Locus Mapping and Genome-Wide Association Studies.* Plos Computational Biology, 2016. **12**(5). 10.1371/journal.pcbi.1004925 PMC4880203 27224861

[36] Sato S (2016). SNP- and haplotype-based genome-wide association studies for growth, carcass, and meat quality traits in a Duroc multigenerational population. BMC Genet.

[37] Yang N (2014). Genome wide association studies using a new nonparametric model reveal the genetic architecture of 17 agronomic traits in an enlarged maize association panel. PLoS Genet.

[38] Schaid DJ, Chen WN, Larson NB (2018). From genome-wide associations to candidate causal variants by statistical fine-mapping. Nat Rev Genet.

[39] Manolio TA (2009). Finding the missing heritability of complex diseases. Nature.

[40] Visscher PM (2012). Five years of GWAS discovery. Am J Hum Genet.

[41] Ahlqvist E (2015). The genetics of diabetic complications. Nat Rev Nephrol.

[42] Altshuler D, Daly MJ, Lander ES (2008). Genetic mapping in human disease. Science.

[43] Marchini J, Howie B (2010). Genotype imputation for genome-wide association studies. Nat Rev Genet.

[44] Huang J (2015). Improved imputation of low-frequency and rare variants using the UK10K haplotype reference panel. Nat Commun.

[45] Fuchsberger C (2016). The genetic architecture of type 2 diabetes. Nature.

[46] Lek M (2016). Analysis of protein-coding genetic variation in 60,706 humans. Nature.

[47] Steinthorsdottir V (2014). Identification of low-frequency and rare sequence variants associated with elevated or reduced risk of type 2 diabetes. Nat Genet.

[48] Fusari CM (2017). Genome-wide association mapping reveals that specific and pleiotropic regulatory mechanisms fine-tune central metabolism and growth in *Arabidopsis*. Plant Cell.

[49] Wu S (2018). Mapping the arabidopsis metabolic landscape by untargeted metabolomics at different environmental conditions. Mol Plant.

[50] Chan EK, Rowe HC, Kliebenstein DJ (2010). Understanding the evolution of defense metabolites in *Arabidopsis thaliana* using genome-wide association mapping. Genetics.

[51] Wu S (2016). Combined use of genome-wide association data and correlation networks unravels key regulators of primary metabolism in *Arabidopsis thaliana*. PLoS Genet.

[52] Exposito-Alonso M (2019). Natural selection on the Arabidopsis thaliana genome in present and future climates. Nature.

[53] Kleessen S (2014). Metabolic efficiency underpins performance trade-offs in growth of *Arabidopsis thaliana*. Nat Commun.

[54] Huang X (2011). Genome-wide association study of flowering time and grain yield traits in a worldwide collection of rice germplasm. Nat Genet.

[55] Wen WW (2014). Metabolome-based genome-wide association study of maize kernel leads to novel biochemical insights. Nat Commun.

[56] Kump KL (2011). Genome-wide association study of quantitative resistance to southern leaf blight in the maize nested association mapping population. Nat Genet.

[57] Chen J (2020). Metabolite-based genome-wide association study enables dissection of the flavonoid decoration pathway of wheat kernels. Plant Biotechnol J.

[58] Zeng X (2020). Genome-wide dissection of co-selected UV-B responsive pathways in the UV-B adaptation of Qingke. Mol Plant.

[59] Hwang EY (2014). A genome-wide association study of seed protein and oil content in soybean. BMC Genomics.

[60] Fang C (2017). Genome-wide association studies dissect the genetic networks underlying agronomical traits in soybean. Genome Biol.

[61] Leamy LJ (2017). A genome-wide association study of seed composition traits in wild soybean (*Glycine soja*). BMC Genomics.

[62] Fang L (2017). Genomic analyses in cotton identify signatures of selection and loci associated with fiber quality and yield traits. Nat Genet.

[63] Wang MJ (2017). Asymmetric subgenome selection and cis-regulatory divergence during cotton domestication. Nat Genet.

[64] Shang Y (2014). Biosynthesis, regulation, and domestication of bitterness in cucumber. Science.

[65] Zhang ZH (2015). Genome-wide mapping of structural variations reveals a copy number variant that determines reproductive morphology in cucumber. Plant Cell.

[66] Wei X (2015). Genetic discovery for oil production and quality in sesame. Nat Commun.

[67] Pandey MK (2014). Genomewide Association Studies for 50 agronomic traits in peanut using the 'Reference set' comprising 300 genotypes from 48 countries of the semi-arid tropics of the world. PLoS ONE.

[68] Cao K (2016). Genome-wide association study of 12 agronomic traits in peach. Nat Commun.

[69] Zhao G (2019). A comprehensive genome variation map of melon identifies multiple domestication events and loci influencing agronomic traits. Nat Genet.

[70] Zhang W (2020). Genome assembly of wild tea tree DASZ reveals pedigree and selection history of tea varieties. Nat Commun.

[71] Zhang L (2017). RNA sequencing provides insights into the evolution of lettuce and the regulation of flavonoid biosynthesis. Nat Commun.

[72] Zhang W (2020). Dissection of the domestication-shaped genetic architecture of lettuce primary metabolism. Plant J.

[73] Janzen GM, Wang L, Hufford MB (2019). The extent of adaptive wild introgression in crops.

[74] Diepenbrock CH (2017). Novel loci underlie natural variation in vitamin E levels in maize grain. Plant Cell.

[75] Li N (2019). Natural variation in ZmFBL41 confers banded leaf and sheath blight resistance in maize. Nat Genet.

[76] Liu W (2017). Genome-wide association mapping reveals a rich genetic architecture of stripe rust resistance loci in emmer wheat (*Triticum turgidum* ssp. dicoccum). Theor Appl Genet.

[77] Joukhadar R (2020). Genome-wide association reveals a complex architecture for rust resistance in 2300 worldwide bread wheat accessions screened under various Australian conditions. Theor Appl Genet.

[78] Sonnewald U (2020). The Cassava Source-Sink project: opportunities and challenges for crop improvement by metabolic engineering. Plant J.

[79] Zhao J (2020). Trait associations in the pangenome of pigeon pea (*Cajanus cajan*). Plant Biotechnol J.

[80] Webber H (2018). Diverging importance of drought stress for maize and winter wheat in Europe. Nat Commun.

[81] Kuroh T (2018). Ethylene-gibberellin signaling underlies adaptation of rice to periodic flooding. Science.

[82] Al-Tamimi N (2016). Salinity tolerance loci revealed in rice using high-throughput non-invasive phenotyping. Nat Commun.

[83] Tang W (2019). Genome-wide associated study identifies NAC42-activated nitrate transporter conferring high nitrogen use efficiency in rice. Nat Commun.

[84] Luo B (2019). Metabolite profiling and genome-wide association studies reveal response mechanisms of phosphorus deficiency in maize seedling. Plant J.

[85] Xiao N (2018). Identification of genes related to cold tolerance and a functional allele that confers cold tolerance. Plant Physiol.

[86] Oladzad A (2019). Single and multi-trait GWAS identify genetic factors associated with production traits in common bean under abiotic stress environments. G3 (Bethesda).

[87] Buckler ES (2009). The genetic architecture of maize flowering time. Science.

[88] Crowell S (2016). Genome-wide association and high-resolution phenotyping link *Oryza sativa* panicle traits to numerous trait-specific QTL clusters. Nat Commun.

[89] Fang C (2017). Genome-wide association studies dissect the genetic networks underlying agronomical traits in soybean. Genome Biol.

[90] Bresadola L (2019). Admixture mapping in interspecific Populus hybrids identifies classes of genomic architectures for phytochemical, morphological and growth traits. New Phytol.

[91] Bararyenya A (2020). Genome-wide association study identified candidate genes controlling continuous storage root formation and bulking in hexaploid sweetpotato. BMC Plant Biol.

[92] Fridman E (2004). Zooming in on a quantitative trait for tomato yield using interspecific introgressions. Science.

[93] Alseekh S (2015). Identification and mode of inheritance of quantitative trait loci for secondary metabolite abundance in tomato. Plant Cell.

[94] Luo J (2015). Metabolite-based genome-wide association studies in plants. Curr Opin Plant Biol.

[95] Alseekh S, Fernie AR (2018). Metabolomics 20years on: what have we learned and what hurdles remain?. Plant J.

[96] Lin H (2009). DWARF27, an Iron-containing protein required for the biosynthesis of strigolactones, regulates rice tiller bud outgrowth. Plant Cell.

[97] Yonekura-Sakakibara K (2014). A flavonoid 3-O-glucoside:2 ''-O-glucosyltransferase responsible for terminal modification of pollen-specific flavonols in Arabidopsis thaliana. Plant J.

[98] Yamamura C (2015). Diterpenoid phytoalexin factor, a bHLH transcription factor, plays a central role in the biosynthesis of diterpenoid phytoalexins in rice. Plant J.

[99] Sadre R (2016). Metabolite diversity in alkaloid biosynthesis: a multilane (diastereomer) highway for camptothecin synthesis in *Camptotheca acuminata*. Plant Cell.

[100] Oliver MJ (2011). A sister group contrast using untargeted global metabolomic analysis delineates the biochemical regulation underlying desiccation tolerance in *Sporobolus stapfianus*. Plant Cell.

[101] Tohge T (2020). Exploiting natural variation in tomato to define pathway structure and metabolic regulation of fruit polyphenolics in the lycopersicum complex. Mol Plant.

[102] Matsuda F (2015). Metabolome-genome-wide association study dissects genetic architecture for generating natural variation in rice secondary metabolism. Plant J.

[103] Zhou S (2019). Metabolome-scale genome-wide association studies reveal chemical diversity and genetic control of maize specialized metabolites. Plant Cell.

[104] Soltis NE, Kliebenstein DJ (2015). Natural variation of plant metabolism: genetic mechanisms, interpretive caveats, and evolutionary and mechanistic insights. Plant Physiol.

[105] Peng M (2017). Differentially evolved glucosyltransferases determine natural variation of rice flavone accumulation and UV-tolerance. Nat Commun.

[106] Li H (2019). Leveraging GWAS data to identify metabolic pathways and networks involved in maize lipid biosynthesis. Plant J.

[107] Burgos E (2020). Validated MAGIC and GWAS populations mapping reveal the link between vitamin E contents and natural variation in chorismate metabolism in tomato. Plant J.

[108] Browning BL, Yu ZX (2009). Simultaneous genotype calling and haplotype phasing improves genotype accuracy and reduces false-positive associations for genome-wide association studies. Am J Hum Genet.

[109] Finno CJ (2014). Risk of false positive genetic associations in complex traits with underlying population structure: a case study. Vet J.

[110] Huang C (2018). ZmCCT9 enhances maize adaptation to higher latitudes. Proc Natl Acad Sci USA.

[111] Li H (2013). Genome-wide association study dissects the genetic architecture of oil biosynthesis in maize kernels. Nat Genet.

[112] Wang Q (2015). Genetic architecture of natural variation in rice chlorophyll content revealed by a genome-wide association study. Mol Plant.

[113] Wen W (2018). An integrated multi-layered analysis of the metabolic networks of different tissues uncovers key genetic components of primary metabolism in maize. Plant J.

[114] Liu J (2017). The conserved and unique genetic architecture of kernel size and weight in maize and rice. Plant Physiol.

[115] Deng M (2017). The genetic architecture of amino acids dissection by association and linkage analysis in maize. Plant Biotechnol J.

[116] Brog YM (2019). A *Solanum neorickii* introgression population providing a powerful complement to the extensively characterized *Solanum pennellii* population. Plant J.

[117] Meng XB (2017). Construction of a genome-wide mutant library in rice using CRISPR/Cas9. Mol Plant.

[118] Lu YM (2017). Genome-wide targeted mutagenesis in rice using the CRISPR/Cas9 system. Mol Plant.

[119] Liu HJ (2020). High-throughput CRISPR/Cas9 mutagenesis streamlines trait gene identification in maize. Plant Cell.

[120] Chen Q (2019). TeoNAM: a nested association mapping population for domestication and agronomic trait analysis in maize. Genetics.

[121] Korte A, Farlow A (2013). The advantages and limitations of trait analysis with GWAS: a review. Plant Methods.

[122] Yang Q (2013). CACTA-like transposable element in ZmCCT attenuated photoperiod sensitivity and accelerated the postdomestication spread of maize. Proc Natl Acad Sci USA.

[123] Huang, X.H. and B. Han (2014) Natural variations and genome-wide association studies in crop plants. In: Merchant SS (ed) Annual review of plant biology*, *vol 65, p 531–551 10.1146/annurev-arplant-050213-035715 24274033

[124] Xing AQ (2015). A rare SNP mutation in Brachytic2 moderately reduces plant height and increases yield potential in maize. J Exp Bot.

[125] Fan CH (2006). GS3, a major QTL for grain length and weight and minor QTL for grain width and thickness in rice, encodes a putative transmembrane protein. Theor Appl Genet.

[126] Xue WY (2008). Natural variation in Ghd7 is an important regulator of heading date and yield potential in rice. Nat Genet.

[127] Lu L (2012). Evolution and association analysis of Ghd7 in rice. PLoS ONE.

[128] Mao HL (2010). Linking differential domain functions of the GS3 protein to natural variation of grain size in rice. Proc Natl Acad Sci USA.

[129] Zhang XJ (2012). Rare allele of OsPPKL1 associated with grain length causes extra-large grain and a significant yield increase in rice. Proc Natl Acad Sci USA.

[130] Ishimaru K (2013). Loss of function of the IAA-glucose hydrolase gene TGW6 enhances rice grain weight and increases yield. Nat Genet.

[131] Zhu CS, Li XR, Yu JM (2011). Integrating rare-variant testing, function prediction, and gene network in composite resequencing-based genome-wide association studies (CR-GWAS). G3.

[132] Listgarten J, Lippert C, Heckerman D (2013). FaST-LMM-Select for addressing confounding from spatial structure and rare variants. Nat Genet.

[133] Kaakinen M (2017). MARV: a tool for genome-wide multi-phenotype analysis of rare variants. Bmc Bioinform.

[134] Dell'Acqua M (2015). Genetic properties of the MAGIC maize population: a new platform for high definition QTL mapping in *Zea mays*. Genome Biol.

[135] Navarro JAR (2017). A study of allelic diversity underlying flowering-time adaptation in maize landraces. Nat Genet.

[136] Wen YJ (2019). An efficient multi-locus mixed model framework for the detection of small and linked QTLs in F-2. Brief Bioinform.

[137] Gibson G (2012). Rare and common variants: twenty arguments. Nat Rev Genet.

[138] Xiao Y (2017). Genome-wide association studies in maize: praise and stargaze. Mol Plant.

[139] Cockram J, Mackay I (2018) Genetic mapping populations for conducting high-resolution trait mapping in plants. In: Varshney RK, Pandey MK, Chitikineni A (eds) Plant genetics and molecular biology, p 109–138 10.1007/10_2017_48 29470600

[140] Atwell S (2010). Genome-wide association study of 107 phenotypes in *Arabidopsis thaliana* inbred lines. Nature.

[141] Kerdaffrec E (2016). Multiple alleles at a single locus control seed dormancy in Swedish Arabidopsis. Elife.

[142] Lin ZW (2012). Parallel domestication of the Shattering1 genes in cereals. Nat Genet.

[143] Lin T (2014). Genomic analyses provide insights into the history of tomato breeding. Nat Genet.

[144] Dickson SP (2010). Rare variants create synthetic genome-wide associations. Plos Biol.

[145] Hayes B (2013). Overview of Statistical Methods for Genome-Wide Association Studies (GWAS). Methods Mol Biol.

[146] Scossa F, Alseekh S, Fernie AR (2021). Integrating multi-omics data for crop improvement. J Plant Physiol.

[147] Ye J (2017). An InDel in the promoter of Al-ACTIVATED MALATE TRANSPORTER9 selected during tomato domestication determines fruit malate contents and aluminum tolerance. Plant Cell.

[148] Chan EK (2010). The complex genetic architecture of the metabolome. PLoS Genet.

[149] Clauw P (2016). Leaf growth response to mild drought: natural variation in Arabidopsis sheds light on trait architecture. Plant Cell.

[150] Li Q (2012). Genome-wide association studies identified three independent polymorphisms associated with α-tocopherol content in maize kernels. PLoS ONE.

[151] Wen W (2016). Combining quantitative genetics approaches with regulatory network analysis to dissect the complex metabolism of the maize kernel. Plant Physiol.

[152] Chen W (2014). Genome-wide association analyses provide genetic and biochemical insights into natural variation in rice metabolism. Nat Genet.

[153] Zhang W (2020). Dissection of the domestication-shaped genetic architecture of lettuce primary metabolism. Plant J.

[154] Wu J (2020). Resequencing of 683 common bean genotypes identifies yield component trait associations across a north-south cline. Nat Genet.

[155] Zhao X (2015). Loci and candidate gene identification for resistance to *Sclerotinia sclerotiorum* in soybean (*Glycine max* L. Merr.) via association and linkage maps. Plant J.

[156] Li W (2017). A natural allele of a transcription factor in rice confers broad-spectrum blast resistance. Cell.

[157] Kuroha T (2018). Ethylene-gibberellin signaling underlies adaptation of rice to periodic flooding. Science.

