# Supplementary material for: Genome-wide association studies: assessing trait characteristics in model and crop plants
Source: Cell Mol Life Sci. 2021 Jul 1;78(15):5743–54. doi: 10.1007/s00018-021-03868-w (PMC8316211; doi:10.1007/s00018-021-03868-w)
Supplement: Supplementary file 1 — Supplementary file1 (DOCX 636 KB) [file 18_2021_3868_MOESM1_ESM.docx]

Supplementary Table: List of genome-wide association studies in plants.

|  | Species [Common Name] | Panel Size [no. of Markers] | Trait [no. of associations] | Reference | Validated (+), not validated (-) |
| --- | --- | --- | --- | --- | --- |
| 1 | *G. hirsutum* [Cotton] | 367 [63058] | Resistance to *Verticillium wilt* and *Fusarium wilt* [28] | [1] | - |
| 2 | *H. vulgare* [Barley] | 3.490 [3072] | Net blotch disease [12] | [2] | - |
| 3 | *O. sativa* [Rice] | 1.132 [NA] | Grain width [25*] | [3] | - |
| 4 | *O. sativa* [Rice] | 305 [2.400.000] | Glycemic Index [10*] | [4] | + |
| 5 | *A. thaliana* [Arabidopsis] | 313 [250.000] | 98 Free amino acids [**] | [5] | + |
| 6 | *A. thaliana* [Arabidopsis] | 360 [250.000] | 19 Free amino acids [**] | [6] | + |
| 7 | *L. perenne* [Ryegrass] | 1.582 [10.878] | *Puccinia coronata f. sp lolli* resistance [NA] | [7] | - |
| 8 | *G. max* [Soybean] | 460 [36.000] | Iron deficiency chlorosis [69] | [8] | - |
| 9 | *A. thaliana* [Arabidopsis] | 199 [250.000] | 107 phenotypes [**] | [9] | - |
| 10 | *P. infestans* [Water Mold] | 1.306 [11.175] | Mycelial Growth, Mefenoxam Resistance, and Mating Type [3] | [10] | - |
| 11 | *A. thaliana* [Arabidopsis] | 360 [250.000] | Fertility under heat stress [4] | [11] | + |
| 12 | *A. thaliana* [Arabidopsis] | 84 [250.000] | Resistance mold disease [2] | [12] | + |
| 13 | *P. abies* [Norway Spruce] | 517 [178.101] | 17 wood related traits [52] | [13] | - |
| 14 | *L. batatas* [Sweetpotato] | 358  [33.068] | Root formation related traits [34] | [14] | - |
| 15 | *Solanum spp.* [Tomato] | 300 [10.000] | 60 fruit metabolites [79] | [15] | + |
| 16 | *J. regia* [Persian Walnut] | 170 [700.000] | Phenological traits and lateral bearing [25] | [16] | - |
| 17 | *M. truncatula* [Medicago] | 179 [5.100.000] | Diseases resistance [2] | [17] | - |
| 18 | *O. sativa* [Rice] | 340 [396.361] | Diseases [bacterial leaf streak and blight] [11] | [18] | - |
| 19 | *A. thaliana* [Arabidopsis] | 227 [1.700.000] | Nutrient deficiencies [87] | [19] | - |
| 20 | *A. thaliana* [Arabidopsis] | 4366 [216.509] | Flowering time [60] | [20] | - |
| 21 | *A. thaliana* [Arabidopsis] | 595 [197.763] | Glucosinolates [474] | [21] | - |
| 22 | *T. Urartu* [Einkorn Wheat] | 298 [75.511] | Diversity [415] | [22] | - |
| 23 | *P. persica* [Peach] | 418 [4.240.000] | Fruit domestication traits [171] | [23] | + |
| 24 | *P. persica* [Peach] | 129 [5.378] | Agronomic traits [**] | [24] | - |
| 25 | *O. sativa* [Rice] | 3000 [404.000] | Transposable elements [26] | [25] | - |
| 26 | *A. thaliana* [Arabidopsis] | 96 [230.000] | Glucosinolate [230] | [26] | + |
| 27 | *A. thaliana* [Arabidopsis] | 96 [200.000 ] | Metabolites [**] | [27] | - |
| 28 | *Solanum spp.* [Tomato] | 180 [5.500.000] | Trichome [**] | [28] | + |
| 29 | *A. thaliana* [Arabidopsis] | 349 [213.000] | Heavy metals-cadmium [12] | [29] | + |
| 30 | *T. aestivum* [Wheat] | 180 [14.646] | Metabolite [1.098] | [30] | + |
| 31 | *Z. mays* [Maize] | 368 [1.250.000] | Transcriptome [19.554] | [31] | + |
| 32 | *Z. mays* [Maize] | 1.257 [51.544] | Agronomic traits [255] | [32] | - |
| 33 | *O. sativa* [Rice] | 529 [6.400.000] | Metabolites [634] | [33] | + |
| 34 | *O. sativa* [Rice] | 502 [3.900.000] | Metabolites [105] | [34] | + |
| 35 | *V. vinifera* [Grape] | 1200 [4.015] | leaf shape [**] | [35] | - |
| 36 | *T. aestivum* [Wheat] | 335 [90.000] | Disease/ Barley yellow virus [36] | [36] | - |
| 37 | *A. thaliana* [Arabidopsis] | 2.029 [3.000.000] | Plant growth under stress [2] | [37] | - |
| 38 | *G. max* [Soybean] | 196 [355.000] | Specialized metabolites, isoflavones [50] | [38] | + |
| 39 | *O. sativa* [Rice] | 373 [3.600.000] | Agronomic traits [**] | [39] | - |
| 40 | *A. thaliana* [Arabidopsis] | 91 [4.000.000] | Leaf growth under mild drought [**] | [40] | - |
| 41 | *H. vulgare* [Barley] | 500 [1.536] | Morphological traits + anthocyanin pigmentation [18* +15*] | [41] | + |
| 42 | *A. thaliana* [Arabidopsis] | 96 [115.301] | Immune system initiation + pathogen resistance [2982 + 3354] | [42] | + |
| 43 | *O. sativa* [Rice] | 242 [700.000] | Panicle and agronomic traits by imaging platforms [10*] | [43] | - |
| 44 | *T. aestivum* [Wheat] | 330 [17.900] | Micro and macronutrient content [279-481] | [44] | - |
| 45 | *M. truncatula* [Medicago] | 226 [6.000.000] | Nodulation [100] | [45] | + |
| 46 | *A. thaliana* [Arabidopsis] | 350 [199.360] | Stress responses drought herbivory fungal infection [20-34; 38-40] | [46] | - |
| 47 | *P. taeda* [Loblolly Pine] | 377 [87.000] | Environmental adaptations [205] | [47] | - |
| 48 | *Z. mays* [Maize] | 153 [1.250.000] | Amino acids 48 traits [247-281] | [48] | + |
| 49 | *A. thaliana* [Arabidopsis] | 333 [250.000] | Salt response roots [**] | [49] | + |
| 50 | *H. vulgare* [Barley] | 180 [703] | Zn accumulation grains [13] | [50] | - |
| 51 | *Z. mays* [Maize] | 4900 [28.900.000] | Vitamin E content grains [1.752] | [51] | - |
| 52 | *Z. mays* [Maize] | 258 [246.477] | Fungal elucidated hormone regulation of diterpenoids [9*] | [52] | + |
| 53 | *O. sativa* [Rice] | 323 [148.934] | Iron stress [23] | [53] | - |
| 54 | *A. thaliana* [Arabidopsis] | 330 [2.800.000] | Stomatal aperture WUE [2] | [54] | - |
| 55 | *O. sativa* [Rice] | 529 [3.916.415] | Morphological trait Flag leaf angle [32-40] | [55] | + |
| 56 | *O. sativa* [Rice] | 529 [3.916.415] | Morphological trait Tiller angle [7] | [56] | + |
| 57 | *O. sativa* [Rice] | 156 [15.400.000] | Specialized metabolites, phenolamindes hydroxycinnamoyl spermidine [26] | [57] | + |
| 58 | *S. indicum* [Sesame] | 400 [1.000.000] | Drought stress [10*] | [58] | + |
| 59 | *H. annuus* [Sunflower] | 288 [5.788] | Flower traits [16] | [59] | - |
| 60 | *G. arboreum* [Cotton] | 215 [1.425.003] | Agronomic traits [98] | [60] | + |
| 61 | *A. thaliana* [Arabidopsis] | 762/211 [879.654] | Drought stress [>1000] | [61] | - |
| 62 | *A. thaliana* [Arabidopsis] | 193/4 [250.000] | Seed mucilage [**] | [62] | + |
| 63 | *R. communis* [Ricinus] | 405 [4.057.720] | Yield associated traits [48-71] | [63] | - |
| 64 | *G. max* [Soybean] | 809 [10.415.168] | Agronomic traits [245*] | [64] | - |
| 65 | *O. sativa* [Rice] | 221 [4.173.185] | mGWAS leaf senescence [8*] | [65] | + |
| 66 | *G. hirsutum* [Cotton] | 258 [1.871.401] | Yield-related traits [119*] | [66] | - |
| 67 | *A. thaliana* [Arabidopsis] | 879 [6.432.557] | Environmental adaptation [**] | [67] | - |
| 68 | *A. thaliana* [Arabidopsis] | 168 [210.000] | Shade avoidance [**] | [68] | - |
| 69 | *A. thaliana* [Arabidopsis] | 157/68 [213.248] | Local adaptation/ environment [based on the top 100 SNPs] | [69] | - |
| 70 | *A. thaliana* [Arabidopsis] | 96 [115.301] | Defense metabolite traits [671] | [70] | - |
| 71 | *O. sativa* [Rice] | 3000 [1.500.000] | Agronomic traits [NA] | [71] | - |
| 72 | *A. thaliana* [Arabidopsis] | 349 [214.051] | Central metabolism and plant growth [131] | [72] | + |
| 73 | *T. durum* [Durum Wheat] | 497 [65.000] | Fungal resistance [1-31] | [73] | - |
| 74 | *A. hypogaea* [Peanut] | 581/496 [11.520/7.672] | Seed and pod weight [19-28 and 11-17] | [74] | - |
| 75 | *H. annuus* [Sunflower] | 239 [609.914] | Resistance to flooding stress [28] | [75] | - |
| 76 | *T. aestivum* [Wheat] | 287 [3.129] | Quality trait [2*-3*] | [76] | - |
| 77 | *A. thaliana* [Arabidopsis] | 96 [178.000] | Root plasticity under nitrogen conditions [53] | [77] | + |
| 78 | *L. japoncus* [Lotus] | 130 [525.800] | Root system architecture [104-3.673] | [78] | + |
| 79 | *O. sativa* [Rice] | 10.074 [1.482.139] | Grain shape and chalkiness traits [12*/11*] | [79] | - |
| 80 | *A. thaliana* [Arabidopsis] | 315 [170.344] | Carotenoid degradation [9] | [80] | + |
| 81 | *Z. mays* [Maize] | 27 [3.300.000] | Genetic Diversity/ geographical adaptation [NA] | [81] | - |
| 82 | *P. persica* [Peach] | 221 [944] | Fruit ripening [1] | [82] | - |
| 83 | *P. trichocarpa* [Black Cottonwood] | 461 [813.000] | Growth, ecophysiology, chemical, metabolomic composition [2-220] | [83] | - |
| 84 | *L. usitatissimum* [Flax] | 200 [674.074] | Seed traits [49-77] | [84] | - |
| 85 | *Z. mays* [Maize] | 209 [NA] | Root architecture under drought stress [62*i] | [85] | - |
| 86 | *T. aestivum* [Wheat] | 210 [7.928] | Floret fertility [218] | [86] | - |
| 87 | *T. aestivum* [Wheat] | 210 [15.696] | Spike development/grain yield [9*] | [87] | - |
| 88 | *T. aestivum* [Wheat] | 210 [15.696] | Plant growth during stem elongation [42] | [88] | - |
| 89 | *O. sativa* [Rice] | 507 [1.959.460] | Image traits under drought [313*-470*] | [89] | + |
| 90 | *A. thaliana* [Arabidopsis] | 104/53 [213.248] | Native/novel environments fitness [1] | [90] | - |
| 91 | *Capsicum spp.* [Pepper] | 208 [109.610] | Capsaicinoid content [69*] | [91] | - |
| 92 | *G. max* [Soybean] | 512 [46.336] | Domestication of gronomic traits [43*] | [92] | - |
| 93 | *L. perenne* [Ryegrass] | 716 [2.034] | Agricultural traits [16] | [93] | - |
| 94 | *P. dactylifera* [Date Palms] | 157 [7.149.205] | Fruit traits [3*] | [94] | + |
| 95 | *H. vulgare* [Barley] | 895 [4.260] | Yield and adaptation [**] | [95] | - |
| 96 | *O. sativa* [Rice] | 175 [3.168] | Specialized metabolites [323] | [96] | - |
| 97 | *Z. mays* [Maize] | 513 [1.250.000] | Primary metabolites [153] | [97] | + |
| 98 | *Z. mays* [Maize] | 368 [1.030.000] | Metabolites [1.459] | [98] | + |
| 99 | *Z. mays* [Maize] | 368 [560.000] | Metabolites [882*] | [99] | - |
| 100 | *Z. mays* [Maize] | 513 [56.110] | Specilaized metabolites [16] | [100] | + |
| 101 | *A. thaliana* [Arabidopsis] | 309 [199.455] | Control and abiotic stress [darkness] [123] | [101] | + |
| 102 | *A. thaliana* [Arabidopsis] | 314 [199.455] | Primary metabolites [117] | [102] | + |
| 103 | *G. hirsutum* [Cotton] | 363/195 [38.822] | Nutritional traits [4*] | [103] | - |
| 104 | *O. sativa* [Rice] | 373 [671.355] | Agronomic traits [37] | [104] | - |
| 105 | *O. sativa* [Rice] | 1.495 [1.654.030] | Agronomic traits [130*] | [105] | - |
| 106 | *O. sativa* [Rice] | 950 [1.345.417] | Agronomic traits [32*] | [106] | - |
| 107 | *M. esculenta* [Cassava] | 173 [114.884] | Carotenoids roots [6] | [107] | - |
| 108 | *A. thaliana* [Arabidopsis] | 81 [206.000] | Flavonoids [8] | [108] | + |
| 109 | *G. hirsutum* [Cotton] | 547 [6.071] | Fiber quality [294] | [109] | + |
| 110 | *S. italic* [Foxtail Millet] | 916 [845.787] | Agronomic traits [512*] | [110] | - |
| 111 | *T. aestivum* [Wheat] | 211 [68.958] | Yield traits N-deficiency [97-159] | [111] | - |
| 112 | *T. aestivum* [Wheat] | 135 [17.372] | Grain yield heterosis [29*] | [112] | - |
| 113 | *H. armigera* [Cotton Bollworm] | 96 [2.097] | Resistance to Bt protein [4] | [113] | + |
| 114 | *H. vulgare* [Barley] | 61 [1.618] | Protein fractions [49] | [114] | - |
| 115 | *T. aestivum* [Wheat] | 2.100 [71.312] | Genome-wide recombination rate variation [**] | [115] | - |
| 116 | *T. aestivum* [Wheat] | 7.887 [6.355] | Grain yield, stress resilience and bread quality [138*] | [116] | - |
| 117 | *A. thaliana* [Arabidopsis] | 347 [20.000] | Root architecture remodeling under salt stress [100*] | [117] | + |
| 118 | *O. sativa* [Rice] | 274 [45.608] | Root morphology under water deficiency [76-106] | [118] | - |
| 119 | *G. max* [Soybean] | 200 [34.680] | Dark green color index [45] | [119] | - |
| 120 | *A. thaliana* [Arabidopsis] | 298 [171.619] | Drought induced ABA accumulation [did lowest top SNPs, 116*] | [120] | + |
| 121 | *A. thaliana* [Arabidopsis] | 211 [214.050] | Litter decomposition [none] | [121] | - |
| 122 | *A. thaliana* [Arabidopsis] | 161 [4.500.000] | Seed dormancy [1*] | [122] | + |
| 123 | *T. durum* [Durum Wheat] | 1.200 [13.000] | Phenology and plant height [11*] | [123] | - |
| 124 | *S. bicolor* [Sorghum] | 196 [3.512.517] | Grain quality [520*] | [124] | - |
| 125 | *A. thaliana* [Arabidopsis] | 223 [216.130] | Zn dependent phosphate homeostasis [13*] | [125] | + |
| 126 | *A. thaliana* [Arabidopsis] | 201 [250.000] | Root meristem zone length [4*] | [126] | - |
| 127 | *A. thaliana* [Arabidopsis] | 350 [214.000] | Aphid resistance [1*] | [127] | + |
| 128 | *A. thaliana* [Arabidopsis] | 349 [214.051] | Morphological traits [4] | [128] | + |
| 129 | *A. thaliana* [Arabidopsis] | 459 [214.051] | Flowering time [41] | [129] | - |
| 130 | *Z. mays* [Maize] | 157 [355.972] | Grey leaf spot resistance [7] | [130] | - |
| 131 | *Z. mays* [Maize] | 5.000 [1.600.000] | Resistance to southern leaf blight [245] | [131] | - |
| 132 | *A. thaliana* [Arabidopsis] | 93 [215.000] | Root length [4*-7*] | [132] | + |
| 133 | *T. aestivum* [Wheat] | 227 [760] | Earliness/flowering [62] | [133] | - |
| 134 | *M. truncatula* [Medicago] | 175 [6.013.644] | Proteomic data [120*-190*] | [134] | + |
| 135 | *T. aestivum* [Wheat] | 94 [17.823] | Mycorrhizal symbiosis [30] | [135] | - |
| 136 | *Z. mays* [Maize] | 369 [1.200.000] | Morphometric diversity shoot apical meristem [51] | [136] | - |
| 137 | *A. thaliana* [Arabidopsis] | 319 [250.000] | Iron toxicity tolerance [1*] | [137] | + |
| 138 | *Z. mays* [Maize] | 464 [9.007.194] | Amylose biosynthesis [27*] | [138] | - |
| 139 | *Z. mays* [Maize] | 368 [560.000] | Lipid biosynthesis [139] | [139] | - |
| 140 | *Z. mays* [Maize] | 500 [560.000] | Heterogeneity to oil-related traits [79] | [140] | + |
| 141 | *Z. mays* [Maize] | 318 [542.438] | *Rhizoctonia solani* resistance [28] | [141] | + |
| 142 | *B. napus* [Rapeseed] | 331 [3.571] | Silique numbers [27*] | [142] | - |
| 143 | *G. hirsutum* [Cotton] | 299 [85.630] | *Verticillium* wilt resistance [17] | [143] | + |
| 144 | *S. bicolor* [Sorghum] | 307 [265.000] | Plant height [8] | [144] | + |
| 145 | *P. persica* [Peach] | 480 [4.980.259] | Agronomic traits [**] | [145] | + |
| 146 | *A. thaliana* [Arabidopsis] | 473 [213.497] | Flowering time [12*] | [146] | - |
| 147 | *G. soya* [Soybean] | 97 [99.085] | Adaptive traits, e.g. FT [17] | [147] | - |
| 148 | *Z. mays* [Maize] | 8.000 [1.000.000] | Flowering time [1.000] | [148] | - |
| 149 | *Z. mays* [Maize] | 332 [56.110] | Ear tips-barrenness, yield associated trait [10*] | [149] | - |
| 150 | *G. hirsutum* [Cotton] | 251 [2.372.767] | Secondary cell wall synthesis [28*] | [150] | - |
| 151 | *Z. mays/ A. thaliana* [Maize/ Arabidopsis] | 277/164 [557.968/208.236] | 260/158 Phenotypic traits [1776*/131*] | [151] | - |
| 152 | *Z. mays* [Maize] | 451 [235.004] | Cuticular conductance [9] | [152] | - |
| 153 | *Z. mays* [Maize] | 281 [591.822] | Tocopherols [34] | [153] | - |
| 154 | *Z. mays* [Maize] | 492 [NA] | 27-kDa γ-zein protein [1*] | [154] | - |
| 155 | *Z. mays* [Maize] | 1404 [11.800.000] | Agronomic traits [355] | [155] | - |
| 156 | *G. max* [Soybean] | 286 [54.294] | Lipid-metabolite related metabolites [334] | [156] | + |
| 157 | *Z. mays* [Maize] | 310 [39.354] | Kernel size [21] | [157] | + |
| 158 | *O. sativa* [Rice] | 584 [700.000] | Magnaporthe oryzae resistance [27*] | [158] | + |
| 159 | *Z. mays* [Maize] | 368 [525.105] | Drought tolerance [107] | [159] | + |
| 160 | *T. aestivum* [Wheat] | 723 [52.303] | Agronomic traits [29*] | [160] | - |
| 161 | *A. thaliana* [Arabidopsis] | 180 [4.500.000] | Regional adaptation [1*] | [161] | - |
| 162 | *Z. mays* [Maize] | 338 [56.110] | Metabolites under low Pi [178] | [162] | + |
| 163 | *G. hirsutum* [Cotton] | 419 [3.660.000] | Fiber related traits [7.383] | [163] | + |
| 164 | *Z. mays* [Maize] | 368 [560.000] | Seedling survival rate under drought [157] | [164] | + |
| 165 | *P. armeniaca* [Apricot] | 72 [56.708] | Plum Pox Virus resistance [5*] | [165] | - |
| 166 | *P. vlugaris* [Common Bean] | 280 [59.884] | Pigments color [2*] | [166] | + |
| 167 | *O. sativa* [Rice] | 1.568 [700.000] | Grain length [2*] | [167] | - |
| 168 | *P. trichocarpa* [Black Cottonwood] | 424 [2.200.000] | Adaxial stomatal traits [280*] | [168] | - |
| 169 | *A. thaliana* [Arabidopsis] | 201 [250.000] | Root development [1*] | [169] | + |
| 170 | *A. thaliana* [Arabidopsis] | 450 [3.000.000] | Temperature on fatty acid desaturation [**] | [170] | - |
| 171 | *T. turgidum* [Durum Wheat] | 311 [30.155] | Agronomic traits [**] | [171] | - |
| 172 | *O. glaberrima* [African Rice] | 93 [199.093] | Salt associated fitness traits [11*] | [172] | - |
| 173 | *A. thaliana* [Arabidopsis] | 166 [250.000] | Secondary growth [1*] | [173] | + |
| 174 | *H. vulgare* [Barley] | 1.000-8.825 [297.550] | Phenotypic traits [11*] | [174] | - |
| 175 | *H. vulgare* [Barley] | 1.862 [3.072] | Malting quality [108/107] | [175] | - |
| 176 | *T. aestivum* [Wheat] | 150 [35.143] | Agronomic traits [94] | [176] | - |
| 177 | *S. bicolor* [Sorghum] | 336 [265.000] | Plant height [**] | [177] | - |
| 178 | *S. bicolor* [Sorghum] | 142 [265.487] | Pigmentation phenotypes [**] | [178] | - |
| 179 | *E. urophylla* [Eucalyptus] | 3.373 [41.320] | Growth traits [**] | [179] | - |
| 180 | *A. thaliana* [Arabidopsis] | 86 [250.000] | Pollen tube reception [2] | [180] | + |
| 181 | *Z. mays* [Maize] | 252 [50.000] | Growth habit [12] | [181] | - |
| 182 | *A. thaliana* [Arabidopsis] | 200 [175.324] | Aluminum and Proton tolerance [140+160] | [182] | - |
| 183 | *C. arietinum* [Chickpea] | 291 [333.001] | SAM morphometric traits [6] | [183] | + |
| 184 | *A. thaliana* [Arabidopsis] | 96 [250.000] | Downy mildew resistance [**] | [184] | - |
| 185 | *G. hirsute/ G. barbadense* [Cotton] | 159/70 [3.876.899] | Agronomic traits [40*/63*] | [185] | - |
| 186 | *Musa spp.* [Banana] | 307 [27.178] | Yield related traits [20] | [186] | - |
| 187 | *M. integrifolia* [Macadamia] | 295 [4.113] | Yield related traits [44] | [187] | - |
| 188 | *Z. mays* [Maize] | 4.998/279 [26.500.000/246.496] | Disease resistance [18*/3*] | [188] | - |
| 189 | *Z. mays* [Maize] | 3.381 [26.500.000] | Hypersensitive response [44*] | [189] | - |
| 190 | *Capsicum spp.* [Pepper] | 383 [51.757] | Carotenoids [1] | [190] | - |
| 191 | *Z. mays* [Maize] | 201 [284.180] | Carotenoids [24] | [191] | - |
| 192 | *Z. mays* [Maize] | 1.651 [268.006] | Kernel color [27] | [192] | - |
| 193 | *Z. mays* [Maize] | 584 [186.849] | Root traits [9 MLM/ 142 GLM] | [193] | - |
| 194 | *Z. mays* [Maize] | 282 [236.205] | Kernel length[0 MLM/12.159 GLM] | [194] | - |
| 195 | *P. vulgaris* [Common Bean] | 238 [382] | Pod indehiscence [4*] | [195] | + |
| 196 | *O. sativa* [Rice] | 281 [2.210.939] | Resistant starch [1*] | [196] | - |
| 197 | *G. max* [Soybean] | 188 [3.343] | Seed quality [18*] | [197] | - |
| 198 | *P. trichocarpa* [Populus] | 334 [29.233] | Wood characteristics [141] | [198] | - |
| 199 | *Capsicum spp.* [Pepper] | 107 [90.794] | *Xanthomonas* resistance [20] | [199] | - |
| 200 | *B. graminis* [Powdery Mildew] | 60 [424.219] | Powdery mildew virulence [1] | [200] | + |
| 201 | *A. thaliana* [Arabidopsis] | 123 [206.000] | *A. brassicae* resistance [42*] | [201] | + |
| 202 | *B. napus* [Canola] | 368 [11.804] | Flowering [142] | [202] | - |
| 203 | *S. lycopersicum* [Tomato] | 295 [23.797.503] | Agricultural traits [NA, only highest SNP for traits indicated] | [203] | - |
| 204 | *A. thaliana* [Arabidopsis] | 198 [8.000.000] | Seed size [38*] | [204] | + |
| 205 | *Z. mays* [Maize] | 281 [376.336] | Inflorescence/ leaf related traits [9*] | [205] | - |
| 206 | *Z. mays* [Maize] | 26 [1.600.000] | Volatile homoterpenes [focused on the lowest p-value SNP] | [206] | + |
| 207 | *Z. mays* [Maize] | 285 [56.110] | Primary metabolites [26] | [207] | - |
| 208 | *A. thaliana* [Arabidopsis] | 192 [250.000] | Root trait upon hormone treatments [114] | [208] | - |
| 209 | *A. thaliana* [Arabidopsis] | 317 [191.362] | Turnip mosaic virus resistance [10*] | [209] | - |
| 210 | *H. vulgare* [Barley] | 1.336 [5.398] | Yield-related salinity tolerance [**] | [210] | - |
| 211 | *H. vulgare* [Barley] | 307 [127.992] | Field-based traits [**] | [211] | - |
| 212 | *A. thaliana* [Arabidopsis] | 139 [NA] | Thermal response [>20*] | [212] | + |
| 213 | *S. cerevisiae* [Yeast] | 165 [282.150] | Toxin tolerance [76] | [213] | + |
| 214 | *Z. mays* [Maize] | 5.000 [30.000.000] | Ionome [**] | [214] | - |
| 215 | *H. vulgare* [Barley] | 1.466 [4.320] | Vitamin E [2] | [215] | - |
| 216 | *Z. mays* [Maize] | 328 [438.161] | Senescence [62] | [216] | + |
| 217 | *L. japonicus* [Lotus] | 136 [525.800] | Environment adapted traits [13] | [217] | - |
| 218 | *S. bicolor* [Sorghum] | 407 [78.012] | Seed element concentration [207-270] | [218] | - |
| 219 | *G. hirsutum/ G. arboreum* [Cotton] | 267 [3.030.326] | Agronomic traits [163*] | [219] | + |
| 220 | *O. sativa* [Rice] | 381 [493.777] | Grain size [4*] | [220] | + |
| 221 | *A. thaliana* [Arabidopsis] | 133/313 [214.052] | Gln related metabolites [21] | [221] | + |
| 222 | *M. sinensis* [Maiden Silvergrass] | 138 [100.000] | Phenotypic traits [35] | [222] | - |
| 223 | *B. cinerea* [Gray Mold] | 96 [237.878] | *B. cinerea* – *A. thaliana* – interaction transcriptome [978.693] | [223] | - |
| 224 | *G. max* [Soybean] | 139 [17.172] | Agronomic traits [15*] | [224] | - |
| 225 | *B. napus* [Rapeseed] | 2.141 [3.971.412/27.216] | Agronomic traits [255/3*] | [225] | - |
| 226 | *O. sativa* [Rice] | 363 [73.147] | Agronomic traits [5*] | [226] | - |
| 227 | *S. bicolor* [Sorghum] | 648 [183.989] | Biomass under drought [213] | [227] | - |
| 228 | *G. max* [Soybean] | 162 [50.000] | Canopy wilting [44*] | [228] | - |
| 229 | *F. excelsior* [Ash tree] | 1.250 [9.347.243] | Resistance to ash dieback fungus [192] | [229] | - |
| 230 | *A. thaliana* [Arabidopsis] | 440 [192.616] | Untargeted metabolites [1*] | [230] | + |
| 231 | *Z. mays* [Maize] | 375 [56.110] | Chilling tolerance [19] | [231] | - |
| 232 | *O. sativa* [Rice] | 510 [4.192.129] | Strigolactone and brassinosteroid signaling [3*] | [232] | + |
| 233 | *Z. mays* [Maize] | 380 [475.821] | Carotenoids [40] | [233] | - |
| 234 | *P. sativum* [Pea] | 135 [16.877] | Heat stress response [32] | [234] | - |
| 235 | *T. Urartu* [wild wheat] | 299 [441.327] | Agronomic traits [25] | [235] | - |
| 236 | *O. sativa* [Rice] | 117 [1.531.224] | NUE-related agronomic traits [7] | [236] | + |
| 237 | *S. bicolor* [Sorghum] | 837/1421 [49.586/18.932] | Grain size [81*] | [237] | - |
| 238 | *H. vulgare* [Barley] | 218 [4.320] | Leaf area and grain numbers [**] | [238] | + |
| 239 | *A. thaliana* [Arabidopsis] | 350 [214.000] | Stress resistance [30*] | [239] | - |
| 240 | *Z. mays* [Maize] | 4.892 [1.600.000] | Leaf architecture [785] | [240] | - |
| 241 | *Solanum spp.* [Tomato] | 398 [2.014.488] | Flavor compounds [251] | [241] | + |
| 242 | *A. thaliana* [Arabidopsis] | 96 [216.130] | Necrosis [15] | [242] | + |
| 243 | *H. vulgare/ T. aestivum* [Barley/Wheat] | 1.317/1.325 [9.000/15.000] | Agronomic traits [14/3 univariate 19/1 multivariate] | [243] | - |
| 244 | *A. thaliana* [Arabidopsis] | 149/948 [250.000] | Adaptation of flowering time [based on top 2.000] | [244] | - |
| 245 | *A. thaliana* [Arabidopsis] | 344 [214.051] | Quantum yield PSII [34*] | [245] | + |
| 246 | *C. cajan* [Pingeonpea] | 292 [446.568] | Agronomic traits [241] | [246] | - |
| 247 | *C. americanus* [Pearl Millet] | 288 [3.117.056] | Agronomic traits arid environments [1.054] | [247] | - |
| 248 | *C. arietinum* [Chickpea] | 429 [3.650.000] | Agronomic, drought and heat related traits [262] | [248] | - |
| 249 | *A. thaliana* [Arabidopsis] | 451 [1.793.606] | Adaptive diversification of growth/ allometric variation [8.250] | [249] | - |
| 250 | *A. thaliana* [Arabidopsis] | 212 [5.094.849] | Cytokinin and angular growth [1*] | [250] | + |
| 251 | *Z. mays* [Maize] | 5.000 [29.700.000] | Phenotypic variation [4.484] | [251] | - |
| 252 | *B. napus* [Rapeseed] | 238 [3.820.000] | Seed quality [72] | [252] | - |
| 253 | *T. aestivum* [Wheat] | 364 [439.209] | Awn/grain length [26] | [253] | + |
| 254 | *O. sativa* [Rice] | 1.122 [4.800.000] | Amylose content [8*] | [254] | - |
| 255 | *Z. mays* [Maize] | 508 [560.000] | Tocopherol related traits [32*] | [255] | + |
| 256 | *O. sativa* [Rice] | 203 [2.300.000] | Agronomic traits [4*] | [256] | - |
| 257 | *B. napus* [Rapeseed] | 520 [31.839] | Seed coat color [22] | [257] | - |
| 258 | *G. hirsutum* [Cotton] | 267 [2.020.834] | Fiber quality [19*] | [258] | - |
| 259 | *O. sativa* [Rice] | 529 [3.916.415] | Chlorophyll content [46*] | [259] | + |
| 260 | *C. sativus* [Cucumber] | 600-700 [24.319] | Horticultural important traits [**] | [260] | - |
| 261 | *A. thaliana* [Arabidopsis] | 133 [243.000] | Environmental stress [25] | [261] | - |
| 262 | *S. lycopersicum* [Tomato] | 195 [2.824.130] | Salt stress ion ratio [9] | [262] | + |
| 263 | *M. sativa* [Alfalfa] | 322 [115.654] | Agronomic traits [42] | [263] | - |
| 264 | *B. napus* [Rapeseed] | 347 [60.000] | *Sclerotinia* resistance [17] | [264] | - |
| 265 | *S. indicum* [Sesame] | 705 [254.781] | Oil production and quality [549*] | [265] | - |
| 266 | *G. hirsutum* [Cotton] | 121 [7.472.949] | Fiber yield and quality [29*] | [266] | + |
| 267 | *B. napus* [Rapeseed] | 991 [2.753.575] | Flowering time [**] | [267] | - |
| 268 | *P. vulgaris* [Common Bean] | 683 [4.811.097] | Yield associated traits [505*] | [268] | - |
| 269 | *T. aestivum* [Wheat] | 410 [660.000] | Stripe rust resistance [35*] | [269] | - |
| 270 | *C. lanatus* [Watermelon] | 1.365 [25.000] | Fruit quality and disease resistance [**] | [270] | - |
| 271 | *Z. mays* [Maize] | 6595/945 [500.000/44.000] | Inflorescence [125*] | [271] | - |
| 272 | *T. aestivum* [Wheat] | 407 [23.371] | Winter hardiness related [10] | [272] | - |
| 273 | *O. sativa* [Rice] | 1.033 [289.231] | Cold stress survival rate [5*] | [273] | + |
| 274 | *S. bicolor* [Sorghum] | 352/219 [82.430/41.997] | Bird feeding behavior [1*] | [274] | - |
| 275 | *O. sativa* [Rice] | 295 [2.767.191] | Grain yield [7*] | [275] | - |
| 276 | *Z. mays* [Maize] | 263 [1.000.000] | Differentially methylated regions CG/CHG/CHH [4336/4096/1426] | [276] | - |
| 277 | *V. unguiculata* [Cowpea] | 299 [500.000] | Pod length [72] | [277] | - |
| 278 | *Z. mays* [Maize] | 600 [940.000] | Kernel row number [145] | [278] | - |
| 279 | *O. sativa* [Rice] | 529 [6.400.000] | Ionomic variation [72*] | [279] | + |
| 280 | *A. thaliana* [Arabidopsis] | 200 [722.000] | Biomass heterosis [750] | [280] | - |
| 281 | *Z. mays* [Maize] | 513 [500.000] | Agronomic traits [10*] | [281] | - |
| 282 | *Z. mays* [Maize] | 368 [557.955] | Photoperiod sensitivity [48] | [282] | - |
| 283 | *O. sativa* [Rice] | 169 [381.007] | Plant architecture [15*] | [283] | + |
| 284 | *O. sativa* [Rice] | 176 [426.337] | Agronomic traits [**] | [284] | + |
| 285 | *B. napus* [Rapeseed] | 203 [24.338] | Oleic acid content [51] | [285] | - |
| 286 | *Solanum spp.* [Tomato] | 302 [4.180.023] | Primary metabolites [388] | [286] | + |
| 287 | *Solanum spp.* [Tomato] | 270 [4.180.023] | Stem diameter [4*] | [287] | + |
| 288 | *Solanum spp.* [Tomato] | 272 [5.500.000] | Fruit malate content [1*] | [288] | + |
| 289 | *T. aestivum* [Wheat] | 142 [4.298] | *P. triticina* and *P. striiformis* resistance [4* and 6*] | [289] | - |
| 290 | *Qingke/ H. vulgare* [Tibetian Hulles Barley/ Barley] | 196 [19.248.055] | Metabolite GWAS [90*] | [290] | + |
| 291 | *G. max* [Soybean] | 219 [201.994] | Seed oil content [110] | [291] | + |
| 292 | *A. hypogaea* [Peanut] | 120 [13.382] | Ionomic content [36*] | [292] | - |
| 293 | *G. max* [Soybean] | 214 [31.914] | Sudden death syndrome [12*] | [293] | - |
| 294 | *G. max* [Soybean] | 313 [31.850] | Quality related traits [87*] | [294] | - |
| 295 | *P. trichocarpa* [Populus] | 917 [8.200.000] | Specialized metabolites [9 + 1*] | [295] | + |
| 296 | *L. sativa* [Lettuce] | 240 [1.100.000] | RNA sequencing + flavonoids [5311* + 6*] | [296] | - |
| 297 | *H. vulgare L. ssp. Spontaneum* [Tibetian wild Barley] | 166 [777] | Drought tolerance [91] | [297] | - |
| 298 | *Z. mays* [Maize] | 513 [11.971.903] | Shoot Na+ content [3*] | [298] | + |
| 299 | *Z. mays* [Maize] | 5.000 [1.600.000] | C and N Metabolites [1.394] | [299] | - |
| 300 | *P. mume* [Mei] | 348 [1.298.196] | Ornamental traits [5*] | [300] | - |
| 301 | *A. thaliana* [Arabidopsis] | 57 [142.048] | Expression data [6.190] | [301] | - |
| 302 | *Z. mays* [Maize] | 663 [90.000.000] | Nodal root number [3*] | [302] | + |
| 303 | *C. melo* [Melon] | 1.067 [2.028.259] | Agronomic trait [208*] | [303] | - |
| 304 | *C. cajan* [Pigeon Pea] | 89 [3.713.723] | Agronomic traits [225] | [304] | - |
| 305 | *Solanum spp.* [Tomato] | 775 [2.316.177] | Flavor [305] | [305] | - |
| 306 | *O. sativa* [Rice] | 312 [183.884] | Cadmium accumulation in grain [14*] | [306] | + |
| 307 | *O. sativa* [Rice] | 413 [44.000] | Complex traits [**] | [307] | - |
| 308 | *G. max* [Soybean] | 330 [25.179] | *S. sclerotiorum* resistance [38] | [308] | - |
| 309 | *Z. mays* [Maize] | 230 [1.250.000] | Arsenic accumulation [45*] | [309] | - |
| 310 | *Z. mays/ S. bicolor* [Maize/Sorghum] | 369/294 [1.200.000/205.000] | Root system architecture [107] | [310] | - |
| 311 | *Z. tritici* [Septoria tritici] | 103 [584.171] | Virulence [1*] | [311] | + |
| 312 | *O. sativa* [Rice] | 533 [6.500.000] | Floral traits [23*] | [312] | + |
| 313 | *Z. mays* [Maize] | 282 [29.000.000] | Untargeted specialized metabolites [**] | [313] | - |
| 314 | *S. bicolor* [Sorghum] | 272 [146.865] | Panicle Architecture [35] | [314] | - |
| 315 | *G. max* [Soybean] | 302 [9.790.744] | Agronomic traits [13*] | [315] | - |
| 316 | *Z. mays* [Maize] | 480 [779.855] | Phenotypic and stem vascular bundles [1.562] | [316] | - |
| 317 | *T. aestivum* [Wheat] | 268 [90.000] | Leaf and stripe rust resistance [22*] | [317] | - |
| 318 | *F. tataricum* [Tartary Buckwheat] | 510 [1.095.748] | Agronomic traits [31] | [318] | + |
| 319 | *A. hypogaea* [Peanut] | 120 [13.382] | Seed composition traits [178*] | [319] | - |
| 320 | *G. max* [Soybean] | 496 [9.357.842] | Nodule number [1*] | [320] | + |
| 321 | *O. sativa* [Rice] | NA [NA] | NUE-related traits [1*] | [321] | + |
| 322 | *G. hirsutum* [Cotton] | 217 [51.060] | Salt tolerance traits [25] | [322] | - |
| 323 | *T. aestivum* [Wheat] | 411 [371.972] | *P. stiiformis* resistance [19*] | [323] | + |
| 324 | *B. napus* [Rapeseed] | 228 [201.817] | Salt tolerance [142] | [324] | - |
| 325 | *T. aestivum* [Wheat] | 143 [6.404] | *P. stiiformis* resistance [17*] | [325] | - |
| 326 | *B. napus* [Rapeseed] | 290 [2.705.480] | Seed oil content [1*] | [326] | - |
| 327 | *P. halepensis* [Mediterranean Pine] | 375 [294] | Growth and leaf pigment/area [23] | [327] | - |
| 328 | *P. dulcis* [Almond] | 149 [93.119] | Nut and seed weight [57] | [328] | - |
| 329 | *T. aestivum* [Wheat] | 486 [90.000] | Quality traits [64*] | [329] | - |
| 330 | *O. sativa* [Rice] | 110 [1.889.946] | tillering response to nitrogen [1*] | [330] | + |
| 331 | *O. sativa* [Rice] | 1.953 [NA] | Rice black-streaked dwarf virus resistance [10*] | [331] | - |
| 332 | *O. sativa* [Rice] | 529 [4.634.871] | Flowering time and photosensitivity [127*] | [332] | - |
| 333 | *O. sativa* [Rice] | 529 [4.634.871] | Neck panicle and flag leaf [135*] | [333] | + |
| 334 | *Z. mays* [Maize] | 513 [490.547] | Grain moisture [71*] | [334] | + |
| 335 | *G. max* [Soybean] | 8.844 [36.489/3.082.234] | Protein and oil content [5-15*] | [335] | - |
| 336 | *H. vulgare* [Barley] | 632 [33.486] | Agronomic traits [1.132] | [336] | - |
| 337 | *B. juncea* [Brown Mustard] | 96 [406.888] | Nitrogen use efficiency [17*] | [337] | - |
| 338 | *G. hirsutum* [Cotton] | 229 [4.476.553] | Yield related traits [119*] | [338] | - |
| 339 | *G. hirsutum* [Cotton] | 550 [473.516] | Drought and salt tolerance [20*] | [339] | - |
| 340 | *O. sativa* [Rice] | 533 [NA] | Oil composition [46*] | [340] | - |
| 341 | *Z. mays* [Maize] | 310 [43.782] | Kernel moisture content [12*] | [341] | - |
| 342 | *Z. mays* [Maize] | 310 [43.782] | Yield related traits [100*] | [342] | + |
| 343 | *L. sativa* [Lettuce] | 189 [16.611] | Primary metabolites [154*] | [343] | + |
| 344 | *O. sativa* [Rice] | 137 [2.000.000] | Root system development [4*] | [344] | - |
| 345 | *H. vulgare* [Barley] | 254 [8.653] | Spikelet development and grain traits [169] | [345] | - |
| 346 | *Z. mays* [Maize] | 508 [560.000] | Fusarium ear rot [34] | [346] | - |
| 347 | *T. aestivum* [Wheat] | 140 [NA] | *P. stiiformis* resistance [12*] | [347] | - |
| 348 | *T. aestivum* [Wheat] | 96 [90.000] | Environmental adaptation [107] | [348] | - |
| 349 | *T. aestivum* [Wheat] | 196 [660.000] | Seminal and nodal root growth under different environments [35*] | [349] | - |
| 350 | *G. max* [Soybean] | 200 [28.926] | Isoflavone content [87] | [350] | + |
| 351 | *A. thaliana* [Arabidopsis] | 102 [250.000] | Flowering time [6*] | [351] | + |
| 352 | *G. arboreum* [Cotton] | 215 [NA] | Fuzz and trichomes [1*] | [352] | + |
| 353 | *Z. mays* [Maize] | 2.240 [297.000/520.000] | Kernel color [24-102] | [353] | - |
| 354 | *G. max/G. soja* [Soybean] | 448/520/429/460 [50.000] | *Phytophtora sojae* resistance [2-7*] | [354] | - |
| 355 | *P. vulgaris* [Common Bean] | 339 [11.870] | Productivity under drought [220*] | [355] | - |
| 356 | *O. sativa* [Rice] | 50 [2.145.095] | Agronomic traits [**] | [356] | - |
| 357 | *T. aestivum* [Wheat] | 221 [90.000] | *B. graminis* resistance [18*] | [357] | - |
| 358 | *G. max* [Soybean] | 137 [2.180.000] | Root system architecture [10*] | [358] | - |
| 359 | *O. sativa* [Rice] | 391 [27.041] | Brown Planthopper resistance [190] | [359] | - |
| 360 | *O. sativa* [Rice] | 389 [5.200.000] | Grain manganese level [**] | [360] | - |
| 361 | *T. turgidum* [Durum Wheat] | 186 [5.933] | Fusarium Head Blight resistance [31*] | [361] | - |
| 362 | *O. sativa* [Rice] | 3024 [6.500.000] | Leaf microbiome [22] | [362] | - |
| 363 | *V. radiata* [Mungbean] | 144 [55.634] | Phosphorus use efficency [136] | [363] | - |
| 364 | *O. sativa* [Rice] | 206 [2.300.000] | Stomatal response in different environmental condiditons [15] | [364] | + |
| 365 | *L. angustifolius* [Lupin] | 126 [2.842] | Agronomic traits [452] | [365] | - |
| 366 | *C. sativa* [Hemp] | 12 [600.000] | Flowering time and sex determination [8*] | [366] | - |
| 367 | *M. esculenta* [Cassava] | 3.354 [27.045] | Hydrogen cyanide [2*] | [367] | + |
| 368 | *A. thaliana* [Arabidopsis] | 165 [13.603] | Vegetation fraction [8*] | [368] | - |
| 369 | *H. vulgare* [Barley] | 882 [9.000] | Low temperature tolerance and vernalization [15*] | [369] | - |
| 370 | *A. thaliana* [Arabidopsis] | 382 [250.000] | Vegetative growth [238] | [370] | - |
| 371 | *O. sativa* [Rice] | 377 [3.883.938] | Panicle number [85-96*] | [371] | + |
| 372 | *C. melo* [Melon] | 297 [2.045.412] | Fruit traits [159] | [372] | - |
| 373 | *Z. mays* [Maize] | 488 [2.668.862] | Phenotypic plasticity [388*] | [373] | - |
| 374 | *G. max* [Soybean] | 286 [54.294] | Seed size and oil content [57] | [374] | + |
| 375 | *T. aestivum* [Wheat] | 1574 [626.245] | *Puccinia triticina* resistance [25*] | [375] | + |
| 376 | *O. sativa* [Rice] | 1.275 [2.081.216] | Agronomic traits [143] | [376] | - |
| 377 | *Z. mays* [Maize] | 526 [155.083] | Popping-related traits [162] | [377] | + |
| 378 | *G. hirsutum* [Cotton] | 200 [2.060.458] | Phenomic data [390*] | [378] | + |
| 379 | *T. aestivum* [Wheat] | 315 [13.450] | Height and stem elongation [2.958] | [379] | - |
| 380 | *Z. mays* [Maize] | 412 [374.540] | North Leaf Blight resistance [5*] | [380] | + |
| 381 | *G. max* [Soybean] | 182 [141.425] | Salt tolerance [11] | [381] | + |
| 382 | *T. aestivum* [Wheat] | 240 [60.000] | *P. striiformis* resistance [12*] | [382] | - |
| 383 | *Z. jujuba* [Chinese Jujube] | 180 [4.651] | Fruit quality [45] | [383] | - |
| 384 | *T. durum* [Durum Wheat] | 384 [10.652] | Flowering time [34*] | [384] | - |
| 385 | *O. sativa* [Rice] | 524 [4.358.600] | Lodging resistance and yield related traits [190*] | [385] | - |
| 386 | *O. sativa* [Rice] | 580 [3.851.692] | Cold adaptation [156*] | [386] | + |
| 387 | *S. bicolor* [Sorghum] | 788 [80.103] | Starch quality and alkali spreading value [61] | [387] | - |
| 388 | *G. hirsutum* [Cotton] | 1.128 [306.814] | Fiber quality and yield-related traits [66*] | [388] | - |
| 389 | *G. max* [Soybean] | 182 [4.000.000] | Carotenoid content [3] | [389] | + |
| 390 | *T. aestivum* [Wheat] | NA [373.106] | Water soluble carbohydrates [275*] | [390] | - |
| 391 | *T. aestivum* [Wheat] | 385 [20.501] | *Puccinia triticina* resistance [96*] | [391] | - |
| 392 | *S. indicum* [Sesame] | 705 [1.000.000] | Root traits [19*] | [392] | + |
| 393 | *V. macrocarpon* [Cranberry] | 282 [21.179] | Fruit-related traits [6] | [393] | - |
| 394 | *P. vulgaris* [Common Bean] | 996 [1.972.528] | Agronomic traits under drought [13*] | [394] | - |
| 395 | *O. sativa* [Rice] | 353 [1.300.000] | Stigma characteristics [41] | [395] | + |
| 396 | *P. vulgaris* [Common Bean] | 192 [49.518] | Micronutrients [12] | [396] | - |
| 397 | *S. oleracea* [Spinach] | 174 [9.783] | Downy Mildew resistance [22] | [397] | - |
| 398 | *M. truncatula* [Medicago] | 175 [6.013.644] | Seedling performance to nitrate supply [45] | [398] | - |
| 399 | *T. aestivum* [Wheat] | 150 [8.526] | Phenotypic traits under elevated ozone [9*] | [399] | - |
| 400 | *P. vulgaris* [Common Bean] | 188 [22.589] | Anthracnose resistance [9] | [400] | - |
| 401 | *Z. mays* [Maize] | 639 [42.667] | Kernel row number [177] | [401] | - |
| 402 | *P. vulgaris* [Common Bean] | 141 [1.927] | Agronomic traits [10] | [402] | - |
| 403 | *S. bicolor* [Sorghum] | 239 [387.672] | Herbicide tolerance [26] | [403] | - |
| 404 | *O. sativa* [Rice] | 67 [2.576] | Blast resistance [36] | [404] | + |
| 405 | *T. turgidum ssp. Dicoccum* [Emmer Wheat] | 176 [5.106] | *P. striiformis* resistance [51*] | [405] | - |
| 406 | *T. aestivum* [Wheat] | 2.300 [49.905] | Rust resistance [161 + 33] | [406] | - |
| 407 | *O. sativa* [Rice] | 553 [304.877] | Salinity tolerance [**] | [407] | - |
| 408 | *O. sativa* [Rice] | 68 [27.192] | Flooding adaptation [6*] | [408] | + |
| 409 | *A. thaliana* [Arabidopsis] | 517 [1.353.386] | Environmental adaptation [6.660] | [409] | + |

*** QTL instead of SNPs**

**** Several associations, not indicated the exact numbers**

**NA not mentioned in the research article**

**References**

1. Abdelraheem, A., et al., *A genome-wide association study uncovers consistent quantitative trait loci for resistance to Verticillium wilt and Fusarium wilt race 4 in the US Upland cotton.* Theor Appl Genet, 2020. **133**(2): p. 563-577.

2. Adhikari, A., et al., *Identification of quantitative trait loci for net form net blotch resistance in contemporary barley breeding germplasm from the USA using genome-wide association mapping.* Theor Appl Genet, 2020. **133**(3): p. 1019-1037.

3. Akakpo, R., et al., *The impact of transposable elements on the structure, evolution and function of the rice genome.* New Phytol, 2020. **226**(1): p. 44-49.

4. Anacleto, R., et al., *Integrating a genome-wide association study with a large-scale transcriptome analysis to predict genetic regions influencing the glycaemic index and texture in rice.* Plant Biotechnol J, 2019. **17**(7): p. 1261-1275.

5. Angelovici, R., et al., *Network-Guided GWAS Improves Identification of Genes Affecting Free Amino Acids.* Plant Physiol, 2017. **173**(1): p. 872-886.

6. Angelovici, R., et al., *Genome-wide analysis of branched-chain amino acid levels in Arabidopsis seeds.* Plant Cell, 2013. **25**(12): p. 4827-43.

7. Arojju, S.K., et al., *Genomic prediction of crown rust resistance in Lolium perenne.* BMC Genet, 2018. **19**(1): p. 35.

8. Assefa, T., et al., *Deconstructing the genetic architecture of iron deficiency chlorosis in soybean using genome-wide approaches.* BMC Plant Biol, 2020. **20**(1): p. 42.

9. Atwell, S., et al., *Genome-wide association study of 107 phenotypes in Arabidopsis thaliana inbred lines.* Nature, 2010. **465**(7298): p. 627-31.

10. Ayala-Usma, D.A., et al., *Genome-Wide Association Study Identifies Single Nucleotide Polymorphism Markers Associated with Mycelial Growth (at 15, 20, and 25°C), Mefenoxam Resistance, and Mating Type in Phytophthora infestans.* Phytopathology, 2020. **110**(4): p. 822-833.

11. Bac-Molenaar, J.A., et al., *Genome-Wide Association Mapping of Fertility Reduction upon Heat Stress Reveals Developmental Stage-Specific QTLs in Arabidopsis thaliana.* Plant Cell, 2015. **27**(7): p. 1857-74.

12. Badet, T., et al., *Parallel evolution of the POQR prolyl oligo peptidase gene conferring plant quantitative disease resistance.* PLoS Genet, 2017. **13**(12): p. e1007143.

13. Baison, J., et al., *Genome-wide association study identified novel candidate loci affecting wood formation in Norway spruce.* Plant J, 2019. **100**(1): p. 83-100.

14. Bararyenya, A., et al., *Genome-wide association study identified candidate genes controlling continuous storage root formation and bulking in hexaploid sweetpotato.* BMC Plant Biol, 2020. **20**(1): p. 3.

15. Bauchet, G., et al., *Identification of major loci and genomic regions controlling acid and volatile content in tomato fruit: implications for flavor improvement.* New Phytol, 2017. **215**(2): p. 624-641.

16. Bernard, A., et al., *Association and linkage mapping to unravel genetic architecture of phenological traits and lateral bearing in Persian walnut (Juglans regia L.).* BMC Genomics, 2020. **21**(1): p. 203.

17. Bonhomme, M., et al., *High-density genome-wide association mapping implicates an F-box encoding gene in Medicago truncatula resistance to Aphanomyces euteiches.* New Phytol, 2014. **201**(4): p. 1328-42.

18. Bossa-Castro, A.M., et al., *Allelic variation for broad-spectrum resistance and susceptibility to bacterial pathogens identified in a rice MAGIC population.* Plant Biotechnol J, 2018. **16**(9): p. 1559-68.

19. Bouain, N., et al., *Systems genomics approaches provide new insights into Arabidopsis thaliana root growth regulation under combinatorial mineral nutrient limitation.* PLoS Genet, 2019. **15**(11): p. e1008392.

20. Brachi, B., et al., *Linkage and association mapping of Arabidopsis thaliana flowering time in nature.* PLoS Genet, 2010. **6**(5): p. e1000940.

21. Brachi, B., et al., *Coselected genes determine adaptive variation in herbivore resistance throughout the native range of Arabidopsis thaliana.* Proc Natl Acad Sci U S A, 2015. **112**(13): p. 4032-7.

22. Brunazzi, A., et al., *Molecular diversity and landscape genomics of the crop wild relative Triticum urartu across the Fertile Crescent.* Plant J, 2018. **94**(4): p. 670-684.

23. Cao, K., et al., *Comparative population genomics identified genomic regions and candidate genes associated with fruit domestication traits in peach.* Plant Biotechnol J, 2019. **17**(10): p. 1954-1970.

24. Cao, K., et al., *Genome-wide association study of 12 agronomic traits in peach.* Nat Commun, 2016. **7**: p. 13246.

25. Carpentier, M.C., et al., *Retrotranspositional landscape of Asian rice revealed by 3000 genomes.* Nat Commun, 2019. **10**(1): p. 24.

26. Chan, E.K., et al., *Combining genome-wide association mapping and transcriptional networks to identify novel genes controlling glucosinolates in Arabidopsis thaliana.* PLoS Biol, 2011. **9**(8): p. e1001125.

27. Chan, E.K., et al., *The complex genetic architecture of the metabolome.* PLoS Genet, 2010. **6**(11): p. e1001198.

28. Chang, J., et al., *Hair, encoding a single C2H2 zinc-finger protein, regulates multicellular trichome formation in tomato.* Plant J, 2018. **96**(1): p. 90-102.

29. Chao, D.Y., et al., *Genome-wide association studies identify heavy metal ATPase3 as the primary determinant of natural variation in leaf cadmium in Arabidopsis thaliana.* PLoS Genet, 2012. **8**(9): p. e1002923.

30. Chen, J., et al., *Metabolite-based genome-wide association study enables dissection of the flavonoid decoration pathway of wheat kernels.* Plant Biotechnol J, 2020.

31. Chen, Q., et al., *Genome-Wide Association Analyses Reveal the Importance of Alternative Splicing in Diversifying Gene Function and Regulating Phenotypic Variation in Maize.* Plant Cell, 2018. **30**(7): p. 1404-1423.

32. Chen, Q., et al., *TeoNAM: A Nested Association Mapping Population for Domestication and Agronomic Trait Analysis in Maize.* Genetics, 2019. **213**(3): p. 1065-1078.

33. Chen, W., et al., *Genome-wide association analyses provide genetic and biochemical insights into natural variation in rice metabolism.* Nat Genet, 2014. **46**(7): p. 714-21.

34. Chen, W., et al., *Comparative and parallel genome-wide association studies for metabolic and agronomic traits in cereals.* Nat Commun, 2016. **7**: p. 12767.

35. Chitwood, D.H., et al., *A modern ampelography: a genetic basis for leaf shape and venation patterning in grape.* Plant Physiol, 2014. **164**(1): p. 259-72.

36. Choudhury, S., et al., *Genome wide association study reveals novel QTL for barley yellow dwarf virus resistance in wheat.* BMC Genomics, 2019. **20**(1): p. 891.

37. Arouisse, B., et al., *Imputation of 3 million SNPs in the Arabidopsis regional mapping population.* Plant J, 2019.

38. Chu, S., et al., *An R2R3-type MYB transcription factor, GmMYB29, regulates isoflavone biosynthesis in soybean.* PLoS Genet, 2017. **13**(5): p. e1006770.

39. Clark, R.M., *Genome-wide association studies coming of age in rice.* Nat Genet, 2010. **42**(11): p. 926-7.

40. Clauw, P., et al., *Leaf Growth Response to Mild Drought: Natural Variation in Arabidopsis Sheds Light on Trait Architecture.* Plant Cell, 2016. **28**(10): p. 2417-2434.

41. Cockram, J., et al., *Genome-wide association mapping to candidate polymorphism resolution in the unsequenced barley genome.* Proc Natl Acad Sci U S A, 2010. **107**(50): p. 21611-6.

42. Corwin, J.A., et al., *The Quantitative Basis of the Arabidopsis Innate Immune System to Endemic Pathogens Depends on Pathogen Genetics.* PLoS Genet, 2016. **12**(2): p. e1005789.

43. Crowell, S., et al., *Genome-wide association and high-resolution phenotyping link Oryza sativa panicle traits to numerous trait-specific QTL clusters.* Nat Commun, 2016. **7**: p. 10527.

44. Cu, S.T., et al., *Genetic dissection of zinc, iron, copper, manganese and phosphorus in wheat (Triticum aestivum L.) grain and rachis at two developmental stages.* Plant Sci, 2020. **291**: p. 110338.

45. Curtin, S.J., et al., *Validating Genome-Wide Association Candidates Controlling Quantitative Variation in Nodulation.* Plant Physiol, 2017. **173**(2): p. 921-931.

46. Davila Olivas, N.H., et al., *Genome-wide association analysis reveals distinct genetic architectures for single and combined stress responses in Arabidopsis thaliana.* New Phytol, 2017. **213**(2): p. 838-851.

47. De La Torre, A.R., B. Wilhite, and D.B. Neale, *Environmental Genome-Wide Association Reveals Climate Adaptation Is Shaped by Subtle to Moderate Allele Frequency Shifts in Loblolly Pine.* Genome Biol Evol, 2019. **11**(10): p. 2976-2989.

48. Deng, M., et al., *The genetic architecture of amino acids dissection by association and linkage analysis in maize.* Plant Biotechnol J, 2017. **15**(10): p. 1250-1263.

49. Deolu-Ajayi, A.O., et al., *Genetic Loci Associated with Early Salt Stress Responses of Roots.* iScience, 2019. **21**: p. 458-473.

50. Detterbeck, A., et al., *The search for candidate genes associated with natural variation of grain Zn accumulation in barley.* Biochem J, 2019. **476**(13): p. 1889-1909.

51. Diepenbrock, C.H., et al., *Novel Loci Underlie Natural Variation in Vitamin E Levels in Maize Grain.* Plant Cell, 2017. **29**(10): p. 2374-2392.

52. Ding, Y., et al., *Multiple genes recruited from hormone pathways partition maize diterpenoid defences.* Nat Plants, 2019. **5**(10): p. 1043-1056.

53. Diop, B., et al., *Bridging old and new: Diversity and evaluation of high iron-associated stress response of rice cultivated in W. Africa.* J Exp Bot, 2020.

54. Dittberner, H., et al., *Natural variation in stomata size contributes to the local adaptation of water-use efficiency in Arabidopsis thaliana.* Mol Ecol, 2018. **27**(20): p. 4052-4065.

55. Dong, H., et al., *Genome-wide association studies reveal that members of bHLH subfamily 16 share a conserved function in regulating flag leaf angle in rice (Oryza sativa).* PLoS Genet, 2018. **14**(4): p. e1007323.

56. Dong, H., et al., *A Novel Tiller Angle Gene, TAC3, together with TAC1 and D2 Largely Determine the Natural Variation of Tiller Angle in Rice Cultivars.* PLoS Genet, 2016. **12**(11): p. e1006412.

57. Dong, X., et al., *Spatio-temporal distribution of phenolamides and the genetics of natural variation of hydroxycinnamoyl spermidine in rice.* Mol Plant, 2014.

58. Dossa, K., et al., *The genetic basis of drought tolerance in the high oil crop Sesamum indicum.* Plant Biotechnol J, 2019. **17**(9): p. 1788-1803.

59. Dowell, J.A., et al., *Genome-Wide Association Mapping of Floral Traits in Cultivated Sunflower (Helianthus annuus).* J Hered, 2019. **110**(3): p. 275-286.

60. Du, X., et al., *Resequencing of 243 diploid cotton accessions based on an updated A genome identifies the genetic basis of key agronomic traits.* Nat Genet, 2018. **50**(6): p. 796-802.

61. Exposito-Alonso, M., et al., *Genomic basis and evolutionary potential for extreme drought adaptation in Arabidopsis thaliana.* Nat Ecol Evol, 2018. **2**(2): p. 352-358.

62. Fabrissin, I., et al., *Natural Variation Reveals a Key Role for Rhamnogalacturonan I in Seed Outer Mucilage and Underlying Genes.* Plant Physiol, 2019. **181**(4): p. 1498-1518.

63. Fan, W., et al., *Sequencing of Chinese castor lines reveals genetic signatures of selection and yield-associated loci.* Nat Commun, 2019. **10**(1): p. 3418.

64. Fang, C., et al., *Genome-wide association studies dissect the genetic networks underlying agronomical traits in soybean.* Genome Biol, 2017. **18**(1): p. 161.

65. Fang, C., et al., *Control of Leaf Senescence by an MeOH-Jasmonates Cascade that Is Epigenetically Regulated by OsSRT1 in Rice.* Mol Plant, 2016. **9**(10): p. 1366-1378.

66. Fang, L., et al., *Genomic analyses in cotton identify signatures of selection and loci associated with fiber quality and yield traits.* Nat Genet, 2017. **49**(7): p. 1089-1098.

67. Ferrero-Serrano, Á. and S.M. Assmann, *Phenotypic and genome-wide association with the local environment of Arabidopsis.* Nat Ecol Evol, 2019. **3**(2): p. 274-285.

68. Filiault, D.L. and J.N. Maloof, *A genome-wide association study identifies variants underlying the Arabidopsis thaliana shade avoidance response.* PLoS Genet, 2012. **8**(3): p. e1002589.

69. Fournier-Level, A., et al., *A map of local adaptation in Arabidopsis thaliana.* Science, 2011. **334**(6052): p. 86-9.

70. Francisco, M., et al., *Genome Wide Association Mapping in Arabidopsis thaliana Identifies Novel Genes Involved in Linking Allyl Glucosinolate to Altered Biomass and Defense.* Front Plant Sci, 2016. **7**: p. 1010.

71. Fuentes, R.R., et al., *Structural variants in 3000 rice genomes.* Genome Res, 2019. **29**(5): p. 870-880.

72. Fusari, C.M., et al., *Genome-Wide Association Mapping Reveals That Specific and Pleiotropic Regulatory Mechanisms Fine-Tune Central Metabolism and Growth in Arabidopsis.* Plant Cell, 2017. **29**(10): p. 2349-2373.

73. Galagedara, N., et al., *Genome-wide association mapping of tan spot resistance in a worldwide collection of durum wheat.* Theor Appl Genet, 2020.

74. Gangurde, S.S., et al., *Nested-association mapping (NAM)-based genetic dissection uncovers candidate genes for seed and pod weights in peanut (Arachis hypogaea).* Plant Biotechnol J, 2019.

75. Gao, L., et al., *Genetic and phenotypic analyses indicate that resistance to flooding stress is uncoupled from performance in cultivated sunflower.* New Phytol, 2019. **223**(3): p. 1657-1670.

76. George, A.W. and C. Cavanagh, *Genome-wide association mapping in plants.* Theor Appl Genet, 2015. **128**(6): p. 1163-74.

77. Gifford, M.L., et al., *Plasticity regulators modulate specific root traits in discrete nitrogen environments.* PLoS Genet, 2013. **9**(9): p. e1003760.

78. Giovannetti, M., et al., *Identification of novel genes involved in phosphate accumulation in Lotus japonicus through Genome Wide Association mapping of root system architecture and anion content.* PLoS Genet, 2019. **15**(12): p. e1008126.

79. Gong, J., et al., *Dissecting the Genetic Basis of Grain Shape and Chalkiness Traits in Hybrid Rice Using Multiple Collaborative Populations.* Mol Plant, 2017. **10**(10): p. 1353-1356.

80. Gonzalez-Jorge, S., et al., *ZEAXANTHIN EPOXIDASE Activity Potentiates Carotenoid Degradation in Maturing Seed.* Plant Physiol, 2016. **171**(3): p. 1837-51.

81. Gore, M.A., et al., *A first-generation haplotype map of maize.* Science, 2009. **326**(5956): p. 1115-7.

82. Guan, L., et al., *Detection and application of genome-wide variations in peach for association and genetic relationship analysis.* BMC Genet, 2019. **20**(1): p. 101.

83. Guerra, F.P., et al., *Exome resequencing and GWAS for growth, ecophysiology, and chemical and metabolomic composition of wood of Populus trichocarpa.* BMC Genomics, 2019. **20**(1): p. 875.

84. Guo, D., et al., *Resequencing 200 Flax Cultivated Accessions Identifies Candidate Genes Related to Seed Size and Weight and Reveals Signatures of Artificial Selection.* Front Plant Sci, 2019. **10**: p. 1682.

85. Guo, J., et al., *Transcriptome and GWAS analyses reveal candidate gene for seminal root length of maize seedlings under drought stress.* Plant Sci, 2020. **292**: p. 110380.

86. Guo, Z., et al., *Genome-wide association analyses of 54 traits identified multiple loci for the determination of floret fertility in wheat.* New Phytol, 2017. **214**(1): p. 257-270.

87. Guo, Z., et al., *Genetic dissection of pre-anthesis sub-phase durations during the reproductive spike development of wheat.* Plant J, 2018.

88. Guo, Z., et al., *Genome-wide association analyses of plant growth traits during the stem elongation phase in wheat.* Plant Biotechnol J, 2018. **16**(12): p. 2042-2052.

89. Guo, Z., et al., *Genome-Wide Association Studies of Image Traits Reveal Genetic Architecture of Drought Resistance in Rice.* Mol Plant, 2018. **11**(6): p. 789-805.

90. Hamilton, J.A., et al., *The role of climate adaptation in colonization success in Arabidopsis thaliana.* Mol Ecol, 2015. **24**(9): p. 2253-63.

91. Han, K., et al., *QTL mapping and GWAS reveal candidate genes controlling capsaicinoid content in Capsicum.* Plant Biotechnol J, 2018. **16**(9): p. 1546-58.

92. Han, Y., et al., *Domestication footprints anchor genomic regions of agronomic importance in soybeans.* New Phytol, 2016. **209**(2): p. 871-84.

93. Harper, J., et al., *Integrating a newly developed BAC-based physical mapping resource for Lolium perenne with a genome-wide association study across a L. perenne European ecotype collection identifies genomic contexts associated with agriculturally important traits.* Ann Bot, 2019. **123**(6): p. 977-992.

94. Hazzouri, K.M., et al., *Genome-wide association mapping of date palm fruit traits.* Nat Commun, 2019. **10**(1): p. 4680.

95. Hill, C.B., et al., *Hybridisation-based target enrichment of phenology genes to dissect the genetic basis of yield and adaptation in barley.* Plant Biotechnol J, 2019. **17**(5): p. 932-944.

96. Matsuda, F., et al., *Metabolome-genome-wide association study dissects genetic architecture for generating natural variation in rice secondary metabolism.* Plant J, 2015. **81**(1): p. 13-23.

97. Wen, W., et al., *An integrated multi-layered analysis of the metabolic networks of different tissues uncovers key genetic components of primary metabolism in maize.* Plant J, 2018. **93**(6): p. 1116-1128.

98. Wen, W., et al., *Metabolome-based genome-wide association study of maize kernel leads to novel biochemical insights.* Nat Commun, 2014. **5**: p. 3438.

99. Wen, W., et al., *Combining Quantitative Genetics Approaches with Regulatory Network Analysis to Dissect the Complex Metabolism of the Maize Kernel.* Plant Physiol, 2016. **170**(1): p. 136-46.

100. Li, Q., et al., *Genome-wide association studies identified three independent polymorphisms associated with α-tocopherol content in maize kernels.* PLoS One, 2012. **7**(5): p. e36807.

101. Wu, S., et al., *Mapping the Arabidopsis Metabolic Landscape by Untargeted Metabolomics at Different Environmental Conditions.* Mol Plant, 2018. **11**(1): p. 118-134.

102. Wu, S., et al., *Combined Use of Genome-Wide Association Data and Correlation Networks Unravels Key Regulators of Primary Metabolism in Arabidopsis thaliana.* PLoS Genet, 2016. **12**(10): p. e1006363.

103. Hinze, L.L., et al., *Diversity analysis of cotton (Gossypium hirsutum L.) germplasm using the CottonSNP63K Array.* BMC Plant Biol, 2017. **17**(1): p. 37.

104. Huang, X., et al., *Genome-wide association studies of 14 agronomic traits in rice landraces.* Nat Genet, 2010. **42**(11): p. 961-7.

105. Huang, X., et al., *Genomic analysis of hybrid rice varieties reveals numerous superior alleles that contribute to heterosis.* Nat Commun, 2015. **6**: p. 6258.

106. Huang, X., et al., *Genome-wide association study of flowering time and grain yield traits in a worldwide collection of rice germplasm.* Nat Genet, 2011. **44**(1): p. 32-9.

107. Ikeogu, U.N., et al., *Genetic Correlation, Genome-Wide Association and Genomic Prediction of Portable NIRS Predicted Carotenoids in Cassava Roots.* Front Plant Sci, 2019. **10**: p. 1570.

108. Ishihara, H., et al., *Natural variation in flavonol accumulation in Arabidopsis is determined by the flavonol glucosyltransferase BGLU6.* J Exp Bot, 2016. **67**(5): p. 1505-17.

109. Islam, M.S., et al., *A MAGIC population-based genome-wide association study reveals functional association of GhRBB1_A07 gene with superior fiber quality in cotton.* BMC Genomics, 2016. **17**(1): p. 903.

110. Jia, G., et al., *A haplotype map of genomic variations and genome-wide association studies of agronomic traits in foxtail millet (Setaria italica).* Nat Genet, 2013. **45**(8): p. 957-61.

111. Jiang, L., et al., *Functional mapping of N deficiency-induced response in wheat yield-component traits by implementing high-throughput phenotyping.* Plant J, 2019. **97**(6): p. 1105-1119.

112. Jiang, Y., et al., *A quantitative genetic framework highlights the role of epistatic effects for grain-yield heterosis in bread wheat.* Nat Genet, 2017. **49**(12): p. 1741-1746.

113. Jin, L., et al., *Dominant point mutation in a tetraspanin gene associated with field-evolved resistance of cotton bollworm to transgenic Bt cotton.* Proc Natl Acad Sci U S A, 2018. **115**(46): p. 11760-11765.

114. Jin, X., K. Wei, and G. Zhang, *A genome-wide association analysis of quantitative trait loci for protein fraction content in Tibetan wild barley.* Biotechnol Lett, 2012. **34**(1): p. 159-65.

115. Jordan, K.W., et al., *The genetic architecture of genome-wide recombination rate variation in allopolyploid wheat revealed by nested association mapping.* Plant J, 2018. **95**(6): p. 1039-1054.

116. Juliana, P., et al., *Improving grain yield, stress resilience and quality of bread wheat using large-scale genomics.* Nat Genet, 2019. **51**(10): p. 1530-1539.

117. Julkowska, M.M., et al., *Genetic Components of Root Architecture Remodeling in Response to Salt Stress.* Plant Cell, 2017. **29**(12): p. 3198-3213.

118. Kadam, N.N., et al., *Genetic Control of Plasticity in Root Morphology and Anatomy of Rice in Response to Water Deficit.* Plant Physiol, 2017. **174**(4): p. 2302-2315.

119. Kaler, A.S., et al., *Genome-Wide Association Mapping of Dark Green Color Index using a Diverse Panel of Soybean Accessions.* Sci Rep, 2020. **10**(1): p. 5166.

120. Kalladan, R., et al., *Natural variation identifies genes affecting drought-induced abscisic acid accumulation in Arabidopsis thaliana.* Proc Natl Acad Sci U S A, 2017. **114**(43): p. 11536-11541.

121. Kazakou, E., et al., *Secondary metabolites have more influence than morphophysiological traits on litter decomposability across genotypes of Arabidopsis thaliana.* New Phytol, 2019. **224**(4): p. 1532-1543.

122. Kerdaffrec, E., et al., *Multiple alleles at a single locus control seed dormancy in Swedish Arabidopsis.* Elife, 2016. **5**.

123. Kidane, Y.G., et al., *A large nested association mapping population for breeding and quantitative trait locus mapping in Ethiopian durum wheat.* Plant Biotechnol J, 2019. **17**(7): p. 1380-1393.

124. Kimani, W., et al., *Genome-wide association study reveals that different pathways contribute to grain quality variation in sorghum (Sorghum bicolor).* BMC Genomics, 2020. **21**(1): p. 112.

125. Kisko, M., et al., *LPCAT1 controls phosphate homeostasis in a zinc-dependent manner.* Elife, 2018. **7**.

126. Klasen, J.R., et al., *A multi-marker association method for genome-wide association studies without the need for population structure correction.* Nat Commun, 2016. **7**: p. 13299.

127. Kloth, K.J., et al., *SIEVE ELEMENT-LINING CHAPERONE1 Restricts Aphid Feeding on Arabidopsis during Heat Stress.* Plant Cell, 2017. **29**(10): p. 2450-2464.

128. Kooke, R., et al., *Genome-Wide Association Mapping and Genomic Prediction Elucidate the Genetic Architecture of Morphological Traits in Arabidopsis.* Plant Physiol, 2016. **170**(4): p. 2187-203.

129. Korte, A., et al., *A mixed-model approach for genome-wide association studies of correlated traits in structured populations.* Nat Genet, 2012. **44**(9): p. 1066-71.

130. Kuki, M.C., et al., *Genome wide association study for gray leaf spot resistance in tropical maize core.* PLoS One, 2018. **13**(6): p. e0199539.

131. Kump, K.L., et al., *Genome-wide association study of quantitative resistance to southern leaf blight in the maize nested association mapping population.* Nat Genet, 2011. **43**(2): p. 163-8.

132. Lachowiec, J., et al., *A Genome-Wide Association Analysis Reveals Epistatic Cancellation of Additive Genetic Variance for Root Length in Arabidopsis thaliana.* PLoS Genet, 2015. **11**(9): p. e1005541.

133. Le Gouis, J., et al., *Genome-wide association analysis to identify chromosomal regions determining components of earliness in wheat.* Theor Appl Genet, 2012. **124**(3): p. 597-611.

134. Le Signor, C., et al., *Genome-wide association studies with proteomics data reveal genes important for synthesis, transport and packaging of globulins in legume seeds.* New Phytol, 2017. **214**(4): p. 1597-1613.

135. Lehnert, H., et al., *Genetics of mycorrhizal symbiosis in winter wheat (Triticum aestivum).* New Phytol, 2017. **215**(2): p. 779-791.

136. Leiboff, S., et al., *Genetic control of morphometric diversity in the maize shoot apical meristem.* Nat Commun, 2015. **6**: p. 8974.

137. Li, B., et al., *GSNOR provides plant tolerance to iron toxicity via preventing iron-dependent nitrosative and oxidative cytotoxicity.* Nat Commun, 2019. **10**(1): p. 3896.

138. Li, C., et al., *The genetic architecture of amylose biosynthesis in maize kernel.* Plant Biotechnol J, 2018. **16**(2): p. 688-695.

139. Li, H., et al., *Leveraging GWAS data to identify metabolic pathways and networks involved in maize lipid biosynthesis.* Plant J, 2019. **98**(5): p. 853-863.

140. Li, H., et al., *Genetic variants and underlying mechanisms influencing variance heterogeneity in maize.* Plant J, 2020.

141. Li, N., et al., *Natural variation in ZmFBL41 confers banded leaf and sheath blight resistance in maize.* Nat Genet, 2019. **51**(10): p. 1540-1548.

142. Li, S., et al., *A systematic dissection of the mechanisms underlying the natural variation of silique number in rapeseed (Brassica napus L.) germplasm.* Plant Biotechnol J, 2020. **18**(2): p. 568-580.

143. Li, T., et al., *Genome-wide association study discovered candidate genes of Verticillium wilt resistance in upland cotton (Gossypium hirsutum L.).* Plant Biotechnol J, 2017. **15**(12): p. 1520-1532.

144. Li, X., et al., *Dissecting repulsion linkage in the dwarfing gene Dw3 region for sorghum plant height provides insights into heterosis.* Proc Natl Acad Sci U S A, 2015. **112**(38): p. 11823-8.

145. Li, Y., et al., *Genomic analyses of an extensive collection of wild and cultivated accessions provide new insights into peach breeding history.* Genome Biol, 2019. **20**(1): p. 36.

146. Li, Y., et al., *Association mapping of local climate-sensitive quantitative trait loci in Arabidopsis thaliana.* Proc Natl Acad Sci U S A, 2010. **107**(49): p. 21199-204.

147. Li, Y.H., et al., *Identification of loci controlling adaptation in Chinese soya bean landraces via a combination of conventional and bioclimatic GWAS.* Plant Biotechnol J, 2020. **18**(2): p. 389-401.

148. Li, Y.X., et al., *Identification of genetic variants associated with maize flowering time using an extremely large multi-genetic background population.* Plant J, 2016. **86**(5): p. 391-402.

149. Li, Z., et al., *Genome-wide association studies and QTL mapping uncover the genetic architecture of ear tip-barrenness in maize.* Physiol Plant, 2020.

150. Li, Z., et al., *Combined GWAS and eQTL analysis uncovers a genetic regulatory network orchestrating the initiation of secondary cell wall development in cotton.* New Phytol, 2020.

151. Liang, Z., Y. Qiu, and J.C. Schnable, *Genome-Phenome Wide Association in Maize and Arabidopsis Identifies a Common Molecular and Evolutionary Signature.* Mol Plant, 2020.

152. Lin, M., et al., *Genome-Wide Association Study for Maize Leaf Cuticular Conductance Identifies Candidate Genes Involved in the Regulation of Cuticle Development.* G3 (Bethesda), 2020.

153. Lipka, A.E., et al., *Genome-wide association study and pathway-level analysis of tocochromanol levels in maize grain.* G3 (Bethesda), 2013. **3**(8): p. 1287-99.

154. Liu, H., et al., *Gene duplication confers enhanced expression of 27-kDa γ-zein for endosperm modification in quality protein maize.* Proc Natl Acad Sci U S A, 2016. **113**(18): p. 4964-9.

155. Liu, H.J., et al., *CUBIC: an atlas of genetic architecture promises directed maize improvement.* Genome Biol, 2020. **21**(1): p. 20.

156. Liu, J.Y., et al., *Three-dimension genetic networks among seed oil-related traits, metabolites and genes reveal the genetic foundations of oil synthesis in soybean.* Plant J, 2020.

157. Liu, M., et al., *Analysis of the genetic architecture of maize kernel size traits by combined linkage and association mapping.* Plant Biotechnol J, 2020. **18**(1): p. 207-221.

158. Liu, M.H., et al., *Genome-wide association study identifies an NLR gene that confers partial resistance to Magnaporthe oryzae in rice.* Plant Biotechnol J, 2019.

159. Liu, S., et al., *Genome-wide analysis of ZmDREB genes and their association with natural variation in drought tolerance at seedling stage of Zea mays L.* PLoS Genet, 2013. **9**(9): p. e1003790.

160. Liu, Y., et al., *A genome-wide association study of 23 agronomic traits in Chinese wheat landraces.* Plant J, 2017. **91**(5): p. 861-873.

161. Long, Q., et al., *Massive genomic variation and strong selection in Arabidopsis thaliana lines from Sweden.* Nat Genet, 2013. **45**(8): p. 884-890.

162. Luo, B., et al., *Metabolite profiling and genome-wide association studies reveal response mechanisms of phosphorus deficiency in maize seedling.* Plant J, 2019. **97**(5): p. 947-969.

163. Ma, Z., et al., *Resequencing a core collection of upland cotton identifies genomic variation and loci influencing fiber quality and yield.* Nat Genet, 2018. **50**(6): p. 803-813.

164. Mao, H., et al., *A transposable element in a NAC gene is associated with drought tolerance in maize seedlings.* Nat Commun, 2015. **6**: p. 8326.

165. Mariette, S., et al., *Genome-wide association links candidate genes to resistance to Plum Pox Virus in apricot (Prunus armeniaca).* New Phytol, 2016. **209**(2): p. 773-84.

166. McClean, P.E., et al., *White seed color in common bean (Phaseolus vulgaris) results from convergent evolution in the P (pigment) gene.* New Phytol, 2018. **219**(3): p. 1112-1123.

167. McCouch, S.R., et al., *Open access resources for genome-wide association mapping in rice.* Nat Commun, 2016. **7**: p. 10532.

168. McKown, A.D., et al., *A role for SPEECHLESS in the integration of leaf stomatal patterning with the growth vs disease trade-off in poplar.* New Phytol, 2019. **223**(4): p. 1888-1903.

169. Meijón, M., et al., *Genome-wide association study using cellular traits identifies a new regulator of root development in Arabidopsis.* Nat Genet, 2014. **46**(1): p. 77-81.

170. Menard, G.N., et al., *Genome Wide Analysis of Fatty Acid Desaturation and Its Response to Temperature.* Plant Physiol, 2017. **173**(3): p. 1594-1605.

171. Mengistu, D.K., et al., *High-density molecular characterization and association mapping in Ethiopian durum wheat landraces reveals high diversity and potential for wheat breeding.* Plant Biotechnol J, 2016. **14**(9): p. 1800-12.

172. Meyer, R.S., et al., *Domestication history and geographical adaptation inferred from a SNP map of African rice.* Nat Genet, 2016. **48**(9): p. 1083-8.

173. Milhinhos, A., et al., *SOBIR1/EVR prevents precocious initiation of fiber differentiation during wood development through a mechanism involving BP and ERECTA.* Proc Natl Acad Sci U S A, 2019. **116**(37): p. 18710-18716.

174. Milner, S.G., et al., *Genebank genomics highlights the diversity of a global barley collection.* Nat Genet, 2019. **51**(2): p. 319-326.

175. Mohammadi, M., et al., *A genome-wide association study of malting quality across eight U.S. barley breeding programs.* Theor Appl Genet, 2015. **128**(4): p. 705-21.

176. Molero, G., et al., *Elucidating the genetic basis of biomass accumulation and radiation use efficiency in spring wheat and its role in yield potential.* Plant Biotechnol J, 2019. **17**(7): p. 1276-1288.

177. Morris, G.P., et al., *Population genomic and genome-wide association studies of agroclimatic traits in sorghum.* Proc Natl Acad Sci U S A, 2013. **110**(2): p. 453-8.

178. Morris, G.P., et al., *Dissecting genome-wide association signals for loss-of-function phenotypes in sorghum flavonoid pigmentation traits.* G3 (Bethesda), 2013. **3**(11): p. 2085-94.

179. Müller, B.S.F., et al., *Independent and Joint-GWAS for growth traits in Eucalyptus by assembling genome-wide data for 3373 individuals across four breeding populations.* New Phytol, 2019. **221**(2): p. 818-833.

180. Müller, L.M., et al., *A subunit of the oligosaccharyltransferase complex is required for interspecific gametophyte recognition in Arabidopsis.* Nat Commun, 2016. **7**: p. 10826.

181. Muraya, M.M., et al., *Genetic variation of growth dynamics in maize (Zea mays L.) revealed through automated non-invasive phenotyping.* Plant J, 2017. **89**(2): p. 366-380.

182. Nakano, Y., et al., *Genome-Wide Association Study and Genomic Prediction Elucidate the Distinct Genetic Architecture of Aluminum and Proton Tolerance in Arabidopsis thaliana.* Front Plant Sci, 2020. **11**: p. 405.

183. Narnoliya, L., et al., *Transcriptional signatures modulating shoot apical meristem morphometric and plant architectural traits enhance yield and productivity in chickpea.* Plant J, 2019. **98**(5): p. 864-883.

184. Nemri, A., et al., *Genome-wide survey of Arabidopsis natural variation in downy mildew resistance using combined association and linkage mapping.* Proc Natl Acad Sci U S A, 2010. **107**(22): p. 10302-7.

185. Nie, X., et al., *High-density genetic variation maps reveal the correlation between asymmetric interspecific introgressions and improvement of agronomic traits in Upland and Pima cotton varieties developed in Xinjiang, China.* Plant J, 2020.

186. Nyine, M., et al., *Association genetics of bunch weight and its component traits in East African highland banana (Musa spp. AAA group).* Theor Appl Genet, 2019. **132**(12): p. 3295-3308.

187. O'Connor, K., et al., *Genome-wide association studies for yield component traits in a macadamia breeding population.* BMC Genomics, 2020. **21**(1): p. 199.

188. Olukolu, B.A., et al., *The Genetics of Leaf Flecking in Maize and Its Relationship to Plant Defense and Disease Resistance.* Plant Physiol, 2016. **172**(3): p. 1787-1803.

189. Olukolu, B.A., et al., *A genome-wide association study of the maize hypersensitive defense response identifies genes that cluster in related pathways.* PLoS Genet, 2014. **10**(8): p. e1004562.

190. Ou, L., et al., *Pan-genome of cultivated pepper (Capsicum) and its use in gene presence-absence variation analyses.* New Phytol, 2018. **220**(2): p. 360-363.

191. Owens, B.F., et al., *A foundation for provitamin A biofortification of maize: genome-wide association and genomic prediction models of carotenoid levels.* Genetics, 2014. **198**(4): p. 1699-716.

192. Owens, B.F., et al., *Genome-Wide Association Study and Pathway-Level Analysis of Kernel Color in Maize.* G3 (Bethesda), 2019. **9**(6): p. 1945-1955.

193. Pace, J., X. Yu, and T. Lübberstedt, *Genomic prediction of seedling root length in maize (Zea mays L.).* Plant J, 2015. **83**(5): p. 903-12.

194. Pang, J., et al., *Kernel size-related genes revealed by an integrated eQTL analysis during early maize kernel development.* Plant J, 2019. **98**(1): p. 19-32.

195. Parker, T.A., et al., *Pod indehiscence is a domestication and aridity resilience trait in common bean.* New Phytol, 2020. **225**(1): p. 558-570.

196. Parween, S., et al., *Balancing the double-edged sword effect of increased resistant starch content and its impact on rice texture: its genetics and molecular physiological mechanisms.* Plant Biotechnol J, 2020.

197. Patil, G., et al., *Dissecting genomic hotspots underlying seed protein, oil, and sucrose content in an interspecific mapping population of soybean using high-density linkage mapping.* Plant Biotechnol J, 2018. **16**(11): p. 1939-1953.

198. Porth, I., et al., *Genome-wide association mapping for wood characteristics in Populus identifies an array of candidate single nucleotide polymorphisms.* New Phytol, 2013. **200**(3): p. 710-26.

199. Potnis, N., et al., *Genome-Wide Association Study of Resistance to Xanthomonas gardneri in the USDA Pepper (Capsicum) Collection.* Phytopathology, 2019. **109**(7): p. 1217-1225.

200. Praz, C.R., et al., *AvrPm2 encodes an RNase-like avirulence effector which is conserved in the two different specialized forms of wheat and rye powdery mildew fungus.* New Phytol, 2017. **213**(3): p. 1301-1314.

201. Rajarammohan, S., et al., *Genome-wide association mapping in Arabidopsis identifies novel genes underlying quantitative disease resistance to Alternaria brassicae.* Mol Plant Pathol, 2018. **19**(7): p. 1719-1732.

202. Raman, H., et al., *GWAS hints at pleiotropic roles for FLOWERING LOCUS T in flowering time and yield-related traits in canola.* BMC Genomics, 2019. **20**(1): p. 636.

203. Razifard, H., et al., *Genomic Evidence for Complex Domestication History of the Cultivated Tomato in Latin America.* Mol Biol Evol, 2020. **37**(4): p. 1118-1132.

204. Ren, D., et al., *A new regulator of seed size control in Arabidopsis identified by a genome-wide association study.* New Phytol, 2019. **222**(2): p. 895-906.

205. Rice, B.R., S.B. Fernandes, and A.E. Lipka, *Multi-Trait Genome-wide Association Studies Reveal Loci Associated with Maize Inflorescence and Leaf Architecture.* Plant Cell Physiol, 2020.

206. Richter, A., et al., *Characterization of Biosynthetic Pathways for the Production of the Volatile Homoterpenes DMNT and TMTT in Zea mays.* Plant Cell, 2016. **28**(10): p. 2651-2665.

207. Riedelsheimer, C., et al., *Genome-wide association mapping of leaf metabolic profiles for dissecting complex traits in maize.* Proc Natl Acad Sci U S A, 2012. **109**(23): p. 8872-7.

208. Ristova, D., et al., *Natural genetic variation shapes root system responses to phytohormones in Arabidopsis.* Plant J, 2018. **96**(2): p. 468-481.

209. Rubio, B., et al., *Genome-wide association study reveals new loci involved in Arabidopsis thaliana and Turnip mosaic virus (TuMV) interactions in the field.* New Phytol, 2019. **221**(4): p. 2026-2038.

210. Saade, S., et al., *Yield-related salinity tolerance traits identified in a nested association mapping (NAM) population of wild barley.* Sci Rep, 2016. **6**: p. 32586.

211. Salas Fernandez, M.G., et al., *A High-Throughput, Field-Based Phenotyping Technology for Tall Biomass Crops.* Plant Physiol, 2017. **174**(4): p. 2008-2022.

212. Sanchez-Bermejo, E., et al., *Genetic Architecture of Natural Variation in Thermal Responses of Arabidopsis.* Plant Physiol, 2015. **169**(1): p. 647-59.

213. Sardi, M., et al., *Genome-wide association across Saccharomyces cerevisiae strains reveals substantial variation in underlying gene requirements for toxin tolerance.* PLoS Genet, 2018. **14**(2): p. e1007217.

214. Schaefer, R.J., et al., *Integrating Coexpression Networks with GWAS to Prioritize Causal Genes in Maize.* Plant Cell, 2018. **30**(12): p. 2922-2942.

215. Schuy, C., et al., *Deciphering the genetic basis for vitamin E accumulation in leaves and grains of different barley accessions.* Sci Rep, 2019. **9**(1): p. 9470.

216. Sekhon, R.S., et al., *Integrated Genome-Scale Analysis Identifies Novel Genes and Networks Underlying Senescence in Maize.* Plant Cell, 2019. **31**(9): p. 1968-1989.

217. Shah, N., et al., *Extreme genetic signatures of local adaptation during Lotus japonicus colonization of Japan.* Nat Commun, 2020. **11**(1): p. 253.

218. Shakoor, N., et al., *Integration of Experiments across Diverse Environments Identifies the Genetic Determinants of Variation in Sorghum bicolor Seed Element Composition.* Plant Physiol, 2016. **170**(4): p. 1989-98.

219. Shen, C., et al., *Population genomics reveals a fine-scale recombination landscape for genetic improvement of cotton.* Plant J, 2019. **99**(3): p. 494-505.

220. Si, L., et al., *OsSPL13 controls grain size in cultivated rice.* Nat Genet, 2016. **48**(4): p. 447-56.

221. Slaten, M.L., et al., *mGWAS Uncovers Gln-Glucosinolate Seed-Specific Interaction and its Role in Metabolic Homeostasis.* Plant Physiol, 2020.

222. Slavov, G.T., et al., *Genome-wide association studies and prediction of 17 traits related to phenology, biomass and cell wall composition in the energy grass Miscanthus sinensis.* New Phytol, 2014. **201**(4): p. 1227-39.

223. Soltis, N.E., et al., *Pathogen Genetic Control of Transcriptome Variation in the Arabidopsis thaliana - Botrytis cinerea Pathosystem.* Genetics, 2020.

224. Sonah, H., et al., *Identification of loci governing eight agronomic traits using a GBS-GWAS approach and validation by QTL mapping in soya bean.* Plant Biotechnol J, 2015. **13**(2): p. 211-21.

225. Song, J.M., et al., *Eight high-quality genomes reveal pan-genome architecture and ecotype differentiation of Brassica napus.* Nat Plants, 2020. **6**(1): p. 34-45.

226. Spindel, J., et al., *Genomic selection and association mapping in rice (Oryza sativa): effect of trait genetic architecture, training population composition, marker number and statistical model on accuracy of rice genomic selection in elite, tropical rice breeding lines.* PLoS Genet, 2015. **11**(2): p. e1004982.

227. Spindel, J.E., et al., *Association mapping by aerial drone reveals 213 genetic associations for Sorghum bicolor biomass traits under drought.* BMC Genomics, 2018. **19**(1): p. 679.

228. Steketee, C.J., et al., *Genome-Wide Association Analyses Reveal Genomic Regions Controlling Canopy Wilting in Soybean.* G3 (Bethesda), 2020. **10**(4): p. 1413-1425.

229. Stocks, J.J., et al., *Genomic basis of European ash tree resistance to ash dieback fungus.* Nat Ecol Evol, 2019. **3**(12): p. 1686-1696.

230. Strauch, R.C., et al., *Discovery of a novel amino acid racemase through exploration of natural variation in Arabidopsis thaliana.* Proc Natl Acad Sci U S A, 2015. **112**(37): p. 11726-31.

231. Strigens, A., et al., *Association mapping for chilling tolerance in elite flint and dent maize inbred lines evaluated in growth chamber and field experiments.* Plant Cell Environ, 2013. **36**(10): p. 1871-87.

232. Sun, S., et al., *Natural selection of a GSK3 determines rice mesocotyl domestication by coordinating strigolactone and brassinosteroid signaling.* Nat Commun, 2018. **9**(1): p. 2523.

233. Suwarno, W.B., et al., *Genome-wide association analysis reveals new targets for carotenoid biofortification in maize.* Theor Appl Genet, 2015. **128**(5): p. 851-64.

234. Tafesse, E.G., et al., *Genome-Wide Association Mapping for Heat Stress Responsive Traits in Field Pea.* Int J Mol Sci, 2020. **21**(6).

235. Talini, R.F., et al., *Genome-wide association study of agronomic and quality traits in a world collection of the wild wheat relative Triticum urartu.* Plant J, 2019.

236. Tang, W., et al., *Genome-wide associated study identifies NAC42-activated nitrate transporter conferring high nitrogen use efficiency in rice.* Nat Commun, 2019. **10**(1): p. 5279.

237. Tao, Y., et al., *Large-scale GWAS in sorghum reveals common genetic control of grain size among cereals.* Plant Biotechnol J, 2020. **18**(4): p. 1093-1105.

238. Thirulogachandar, V., et al., *Leaf primordium size specifies leaf width and vein number among row-type classes in barley.* Plant J, 2017. **91**(4): p. 601-612.

239. Thoen, M.P., et al., *Genetic architecture of plant stress resistance: multi-trait genome-wide association mapping.* New Phytol, 2017. **213**(3): p. 1346-1362.

240. Tian, F., et al., *Genome-wide association study of leaf architecture in the maize nested association mapping population.* Nat Genet, 2011. **43**(2): p. 159-62.

241. Tieman, D., et al., *A chemical genetic roadmap to improved tomato flavor.* Science, 2017. **355**(6323): p. 391-394.

242. Todesco, M., et al., *Natural allelic variation underlying a major fitness trade-off in Arabidopsis thaliana.* Nature, 2010. **465**(7298): p. 632-6.

243. Tsai, H.Y., et al., *Genomic prediction and GWAS of yield, quality and disease-related traits in spring barley and winter wheat.* Sci Rep, 2020. **10**(1): p. 3347.

244. van Heerwaarden, J., M. van Zanten, and W. Kruijer, *Genome-Wide Association Analysis of Adaptation Using Environmentally Predicted Traits.* PLoS Genet, 2015. **11**(10): p. e1005594.

245. van Rooijen, R., et al., *Natural variation of YELLOW SEEDLING1 affects photosynthetic acclimation of Arabidopsis thaliana.* Nat Commun, 2017. **8**(1): p. 1421.

246. Varshney, R.K., et al., *Whole-genome resequencing of 292 pigeonpea accessions identifies genomic regions associated with domestication and agronomic traits.* Nat Genet, 2017. **49**(7): p. 1082-1088.

247. Varshney, R.K., et al., *Pearl millet genome sequence provides a resource to improve agronomic traits in arid environments.* Nat Biotechnol, 2017. **35**(10): p. 969-976.

248. Varshney, R.K., et al., *Resequencing of 429 chickpea accessions from 45 countries provides insights into genome diversity, domestication and agronomic traits.* Nat Genet, 2019. **51**(5): p. 857-864.

249. Vasseur, F., et al., *Adaptive diversification of growth allometry in the plant Arabidopsis thaliana.* Proc Natl Acad Sci U S A, 2018. **115**(13): p. 3416-3421.

250. Waidmann, S., et al., *Cytokinin functions as an asymmetric and anti-gravitropic signal in lateral roots.* Nat Commun, 2019. **10**(1): p. 3540.

251. Wallace, J.G., et al., *Association mapping across numerous traits reveals patterns of functional variation in maize.* PLoS Genet, 2014. **10**(12): p. e1004845.

252. Wang, B., et al., *Dissection of the genetic architecture of three seed-quality traits and consequences for breeding in Brassica napus.* Plant Biotechnol J, 2018. **16**(7): p. 1336-1348.

253. Wang, D., et al., *Natural variations in the promoter of Awn Length Inhibitor 1 (ALI-1) are associated with awn elongation and grain length in common wheat.* Plant J, 2020. **101**(5): p. 1075-1090.

254. Wang, D.R., et al., *An imputation platform to enhance integration of rice genetic resources.* Nat Commun, 2018. **9**(1): p. 3519.

255. Wang, H., et al., *Beyond pathways: genetic dissection of tocopherol content in maize kernels by combining linkage and association analyses.* Plant Biotechnol J, 2018. **16**(8): p. 1464-1475.

256. Wang, H., et al., *The Power of Inbreeding: NGS-Based GWAS of Rice Reveals Convergent Evolution during Rice Domestication.* Mol Plant, 2016. **9**(7): p. 975-85.

257. Wang, J., et al., *Genome-Wide Association Mapping of Seed Coat Color in Brassica napus.* J Agric Food Chem, 2017. **65**(26): p. 5229-5237.

258. Wang, M., et al., *Asymmetric subgenome selection and cis-regulatory divergence during cotton domestication.* Nat Genet, 2017. **49**(4): p. 579-587.

259. Wang, Q., et al., *Genetic Architecture of Natural Variation in Rice Chlorophyll Content Revealed by a Genome-Wide Association Study.* Mol Plant, 2015. **8**(6): p. 946-57.

260. Wang, X., et al., *The USDA cucumber (Cucumis sativus L.) collection: genetic diversity, population structure, genome-wide association studies, and core collection development.* Hortic Res, 2018. **5**: p. 64.

261. Wang, X., et al., *Cis-regulated alternative splicing divergence and its potential contribution to environmental responses in Arabidopsis.* Plant J, 2019. **97**(3): p. 555-570.

262. Wang, Z., et al., *Loss of salt tolerance during tomato domestication conferred by variation in a Na(+) /K(+) transporter.* Embo j, 2020: p. e103256.

263. Wang, Z., et al., *A genome-wide association study approach to the identification of candidate genes underlying agronomic traits in alfalfa (Medicago sativa L.).* Plant Biotechnol J, 2020. **18**(3): p. 611-613.

264. Wei, L., et al., *Genome-wide association analysis and differential expression analysis of resistance to Sclerotinia stem rot in Brassica napus.* Plant Biotechnol J, 2016. **14**(6): p. 1368-80.

265. Wei, X., et al., *Genetic discovery for oil production and quality in sesame.* Nat Commun, 2015. **6**: p. 8609.

266. Wen, T., et al., *Linkage and association mapping reveals the genetic basis of brown fibre (Gossypium hirsutum).* Plant Biotechnol J, 2018. **16**(9): p. 1654-66.

267. Wu, D., et al., *Whole-Genome Resequencing of a Worldwide Collection of Rapeseed Accessions Reveals the Genetic Basis of Ecotype Divergence.* Mol Plant, 2019. **12**(1): p. 30-43.

268. Wu, J., et al., *Resequencing of 683 common bean genotypes identifies yield component trait associations across a north-south cline.* Nat Genet, 2020. **52**(1): p. 118-125.

269. Wu, J., et al., *Association Analysis Identifies New Loci for Resistance to Chinese Yr26-Virulent Races of the Stripe Rust Pathogen in a Diverse Panel of Wheat Germplasm.* Plant Dis, 2020: p. Pdis12192663re.

270. Wu, S., et al., *Genome of 'Charleston Gray', the principal American watermelon cultivar, and genetic characterization of 1,365 accessions in the U.S. National Plant Germplasm System watermelon collection.* Plant Biotechnol J, 2019. **17**(12): p. 2246-2258.

271. Wu, X., et al., *Joint-linkage mapping and GWAS reveal extensive genetic loci that regulate male inflorescence size in maize.* Plant Biotechnol J, 2016. **14**(7): p. 1551-62.

272. Würschum, T., et al., *Copy number variations of CBF genes at the Fr-A2 locus are essential components of winter hardiness in wheat.* Plant J, 2017. **89**(4): p. 764-773.

273. Xiao, N., et al., *Identification of Genes Related to Cold Tolerance and a Functional Allele That Confers Cold Tolerance.* Plant Physiol, 2018. **177**(3): p. 1108-1123.

274. Xie, P., et al., *Control of Bird Feeding Behavior by Tannin1 through Modulating the Biosynthesis of Polyphenols and Fatty Acid-Derived Volatiles in Sorghum.* Mol Plant, 2019. **12**(10): p. 1315-1324.

275. Xie, W., et al., *Breeding signatures of rice improvement revealed by a genomic variation map from a large germplasm collection.* Proc Natl Acad Sci U S A, 2015. **112**(39): p. E5411-9.

276. Xu, J., et al., *Population-level analysis reveals the widespread occurrence and phenotypic consequence of DNA methylation variation not tagged by genetic variation in maize.* Genome Biol, 2019. **20**(1): p. 243.

277. Xu, P., et al., *Genomic regions, cellular components and gene regulatory basis underlying pod length variations in cowpea (V. unguiculata L. Walp).* Plant Biotechnol J, 2017. **15**(5): p. 547-557.

278. Yang, J., et al., *Extreme-phenotype genome-wide association study (XP-GWAS): a method for identifying trait-associated variants by sequencing pools of individuals selected from a diversity panel.* Plant J, 2015. **84**(3): p. 587-96.

279. Yang, M., et al., *Genome-Wide Association Studies Reveal the Genetic Basis of Ionomic Variation in Rice.* Plant Cell, 2018. **30**(11): p. 2720-2740.

280. Yang, M., et al., *Genomic architecture of biomass heterosis in Arabidopsis.* Proc Natl Acad Sci U S A, 2017. **114**(30): p. 8101-8106.

281. Yang, N., et al., *Genome wide association studies using a new nonparametric model reveal the genetic architecture of 17 agronomic traits in an enlarged maize association panel.* PLoS Genet, 2014. **10**(9): p. e1004573.

282. Yang, Q., et al., *CACTA-like transposable element in ZmCCT attenuated photoperiod sensitivity and accelerated the postdomestication spread of maize.* Proc Natl Acad Sci U S A, 2013. **110**(42): p. 16969-74.

283. Yano, K., et al., *GWAS with principal component analysis identifies a gene comprehensively controlling rice architecture.* Proc Natl Acad Sci U S A, 2019. **116**(42): p. 21262-21267.

284. Yano, K., et al., *Genome-wide association study using whole-genome sequencing rapidly identifies new genes influencing agronomic traits in rice.* Nat Genet, 2016. **48**(8): p. 927-34.

285. Yao, M., et al., *GWAS and co-expression network combination uncovers multigenes with close linkage effects on the oleic acid content accumulation in Brassica napus.* BMC Genomics, 2020. **21**(1): p. 320.

286. Ye, J., et al., *Genome-wide association analysis identifies a natural variation in basic helix-loop-helix transcription factor regulating ascorbate biosynthesis via D-mannose/L-galactose pathway in tomato.* PLoS Genet, 2019. **15**(5): p. e1008149.

287. Ye, J., et al., *Tomato SD1, encoding a kinase interacting protein, is a major locus controlling stem development.* J Exp Bot, 2020.

288. Ye, J., et al., *An InDel in the Promoter of Al-ACTIVATED MALATE TRANSPORTER9 Selected during Tomato Domestication Determines Fruit Malate Contents and Aluminum Tolerance.* Plant Cell, 2017. **29**(9): p. 2249-2268.

289. Yuan, C., et al., *Genome-Wide Mapping of Adult Plant Resistance to Leaf Rust and Stripe Rust in CIMMYT Wheat Line Arableu#1.* Plant Dis, 2020: p. Pdis10192198re.

290. Zeng, X., et al., *Genome-wide Dissection of Co-selected UV-B Responsive Pathways in the UV-B Adaptation of Qingke.* Mol Plant, 2020. **13**(1): p. 112-127.

291. Zhang, D., et al., *Artificial selection on GmOLEO1 contributes to the increase in seed oil during soybean domestication.* PLoS Genet, 2019. **15**(7): p. e1008267.

292. Zhang, H., et al., *GWAS and Coexpression Network Reveal Ionomic Variation in Cultivated Peanut.* J Agric Food Chem, 2019. **67**(43): p. 12026-12036.

293. Zhang, J., et al., *Genome-wide association and epistasis studies unravel the genetic architecture of sudden death syndrome resistance in soybean.* Plant J, 2015. **84**(6): p. 1124-36.

294. Zhang, J., et al., *Genome-wide Scan for Seed Composition Provides Insights into Soybean Quality Improvement and the Impacts of Domestication and Breeding.* Mol Plant, 2018. **11**(3): p. 460-472.

295. Zhang, J., et al., *Genome-wide association studies and expression-based quantitative trait loci analyses reveal roles of HCT2 in caffeoylquinic acid biosynthesis and its regulation by defense-responsive transcription factors in Populus.* New Phytol, 2018. **220**(2): p. 502-516.

296. Zhang, L., et al., *RNA sequencing provides insights into the evolution of lettuce and the regulation of flavonoid biosynthesis.* Nat Commun, 2017. **8**(1): p. 2264.

297. Zhang, M., et al., *Response of Tibetan Wild Barley Genotypes to Drought Stress and Identification of Quantitative Trait Loci by Genome-Wide Association Analysis.* Int J Mol Sci, 2019. **20**(3).

298. Zhang, M., et al., *A HAK family Na(+) transporter confers natural variation of salt tolerance in maize.* Nat Plants, 2019. **5**(12): p. 1297-1308.

299. Zhang, N., et al., *Genome-wide association of carbon and nitrogen metabolism in the maize nested association mapping population.* Plant Physiol, 2015. **168**(2): p. 575-83.

300. Zhang, Q., et al., *The genetic architecture of floral traits in the woody plant Prunus mume.* Nat Commun, 2018. **9**(1): p. 1702.

301. Zhang, X., A.J. Cal, and J.O. Borevitz, *Genetic architecture of regulatory variation in Arabidopsis thaliana.* Genome Res, 2011. **21**(5): p. 725-33.

302. Zhang, Z., et al., *The genetic architecture of nodal root number in maize.* Plant J, 2018. **93**(6): p. 1032-1044.

303. Zhao, G., et al., *A comprehensive genome variation map of melon identifies multiple domestication events and loci influencing agronomic traits.* Nat Genet, 2019. **51**(11): p. 1607-1615.

304. Zhao, J., et al., *Trait associations in the pangenome of pigeon pea (Cajanus cajan).* Plant Biotechnol J, 2020.

305. Zhao, J., et al., *Meta-analysis of genome-wide association studies provides insights into genetic control of tomato flavor.* Nat Commun, 2019. **10**(1): p. 1534.

306. Zhao, J., et al., *Genome-wide association study and candidate gene analysis of rice cadmium accumulation in grain in a diverse rice collection.* Rice (N Y), 2018. **11**(1): p. 61.

307. Zhao, K., et al., *Genome-wide association mapping reveals a rich genetic architecture of complex traits in Oryza sativa.* Nat Commun, 2011. **2**: p. 467.

308. Zhao, X., et al., *Loci and candidate gene identification for resistance to Sclerotinia sclerotiorum in soybean (Glycine max L. Merr.) via association and linkage maps.* Plant J, 2015. **82**(2): p. 245-55.

309. Zhao, Z., et al., *Genetic-based dissection of arsenic accumulation in maize using a genome-wide association analysis method.* Plant Biotechnol J, 2018. **16**(5): p. 1085-1093.

310. Zheng, Z., et al., *Shared Genetic Control of Root System Architecture between Zea mays and Sorghum bicolor.* Plant Physiol, 2020. **182**(2): p. 977-991.

311. Zhong, Z., et al., *A small secreted protein in Zymoseptoria tritici is responsible for avirulence on wheat cultivars carrying the Stb6 resistance gene.* New Phytol, 2017. **214**(2): p. 619-631.

312. Zhou, H., et al., *Genome-wide Association Analyses Reveal the Genetic Basis of Stigma Exsertion in Rice.* Mol Plant, 2017. **10**(4): p. 634-644.

313. Zhou, S., et al., *Metabolome-Scale Genome-Wide Association Studies Reveal Chemical Diversity and Genetic Control of Maize Specialized Metabolites.* Plant Cell, 2019. **31**(5): p. 937-955.

314. Zhou, Y., et al., *Semiautomated Feature Extraction from RGB Images for Sorghum Panicle Architecture GWAS.* Plant Physiol, 2019. **179**(1): p. 24-37.

315. Zhou, Z., et al., *Resequencing 302 wild and cultivated accessions identifies genes related to domestication and improvement in soybean.* Nat Biotechnol, 2015. **33**(4): p. 408-14.

316. Zhang, Y., et al., *Dissecting the phenotypic components and genetic architecture of maize stem vascular bundles using high-throughput phenotypic analysis.* Plant Biotechnol J, 2021. **19**(1): p. 35-50.

317. Zhang, P., et al., *Genome-wide association mapping of leaf rust and stripe rust resistance in wheat accessions using the 90K SNP array.* Theor Appl Genet, 2021.

318. Zhang, K., et al., *Resequencing of global Tartary buckwheat accessions reveals multiple domestication events and key loci associated with agronomic traits.* Genome Biol, 2021. **22**(1): p. 23.

319. Zhang, H., et al., *Identification of potential QTLs and genes associated with seed composition traits in peanut (Arachis hypogaea L.) using GWAS and RNA-Seq analysis.* Gene, 2021. **769**: p. 145215.

320. Zhang, B., et al., *Glycine max NNL1 restricts symbiotic compatibility with widely distributed bradyrhizobia via root hair infection.* Nat Plants, 2021. **7**(1): p. 73-86.

321. Yu, J., et al., *Enhanced OsNLP4-OsNiR cascade confers nitrogen use efficiency by promoting tiller number in rice.* Plant Biotechnol J, 2021. **19**(1): p. 167-176.

322. Xu, P., et al., *Genome-wide association analysis reveals genetic variations and candidate genes associated with salt tolerance related traits in Gossypium hirsutum.* BMC Genomics, 2021. **22**(1): p. 26.

323. Wu, J., et al., *A large-scale genomic association analysis identifies the candidate causal genes conferring stripe rust resistance under multiple field environments.* Plant Biotechnol J, 2021. **19**(1): p. 177-191.

324. Wassan, G.M., et al., *Identification of genetic variation for salt tolerance in Brassica napus using genome-wide association mapping.* Mol Genet Genomics, 2021.

325. Wang, Y., et al., *Genome-wide association mapping reveals potential novel loci controlling stripe rust resistance in a Chinese wheat landrace diversity panel from the southern autumn-sown spring wheat zone.* BMC Genomics, 2021. **22**(1): p. 34.

326. Wang, H., et al., *Genome-wide association study reveals a patatin-like lipase relating to the reduction of seed oil content in Brassica napus.* BMC Plant Biol, 2021. **21**(1): p. 6.

327. Santini, F., et al., *Bridging the genotype-phenotype gap for a Mediterranean pine by semi-automatic crown identification and multispectral imagery.* New Phytol, 2021. **229**(1): p. 245-258.

328. Pavan, S., et al., *Almond diversity and homozygosity define structure, kinship, inbreeding, and linkage disequilibrium in cultivated germplasm, and reveal genomic associations with nut and seed weight.* Hortic Res, 2021. **8**(1): p. 15.

329. Lou, H., et al., *Genome-wide association study of six quality-related traits in common wheat (Triticum aestivum L.) under two sowing conditions.* Theor Appl Genet, 2021. **134**(1): p. 399-418.

330. Liu, Y., et al., *Genomic basis of geographical adaptation to soil nitrogen in rice.* Nature, 2021.

331. Liu, Q., et al., *Genome-Wide Association Study on Resistance to Rice Black-Streaked Dwarf Disease Caused by Rice black-streaked dwarf virus.* Plant Dis, 2021: p. Pdis10192263re.

332. Liu, C., et al., *Genome-wide association study of flowering time reveals complex genetic heterogeneity and epistatic interactions in rice.* Gene, 2021. **770**: p. 145353.

333. Liao, S., et al., *Genetic basis of vascular bundle variations in rice revealed by genome-wide association study.* Plant Sci, 2021. **302**: p. 110715.

334. Li, W., et al., *The genetic architecture of the dynamic changes in grain moisture in maize.* Plant Biotechnol J, 2021.

335. Kim, M.S., et al., *The patterns of deleterious mutations during the domestication of soybean.* Nat Commun, 2021. **12**(1): p. 97.

336. Hill, C.B., et al., *A global barley panel revealing genomic signatures of breeding in modern Australian cultivars.* Plant J, 2021.

337. Gupta, N., et al., *Association genetics of the parameters related to nitrogen use efficiency in Brassica juncea L.* Plant Mol Biol, 2021. **105**(1-2): p. 161-175.

338. Fang, L., et al., *Divergent improvement of two cultivated allotetraploid cotton species.* Plant Biotechnol J, 2021.

339. Abdelraheem, A., et al., *GWAS reveals consistent QTL for drought and salt tolerance in a MAGIC population of 550 lines derived from intermating of 11 Upland cotton (Gossypium hirsutum) parents.* Mol Genet Genomics, 2021. **296**(1): p. 119-129.

340. Zhou, H., et al., *Genetic architecture and key genes controlling the diversity of oil composition in rice grains.* Mol Plant, 2020.

341. Zhang, Y., et al., *Combined linkage mapping and association analysis reveals genetic control of maize kernel moisture content.* Physiol Plant, 2020. **170**(4): p. 508-518.

342. Zhang, X., et al., *A combination of linkage mapping and GWAS brings new elements on the genetic basis of yield-related traits in maize across multiple environments.* Theor Appl Genet, 2020. **133**(10): p. 2881-2895.

343. Zhang, W., et al., *Dissection of the domestication-shaped genetic architecture of lettuce primary metabolism.* Plant J, 2020. **104**(3): p. 613-630.

344. Zhang, H., et al., *Genome-Wide Association Study of Root System Development at Seedling Stage in Rice.* Genes (Basel), 2020. **11**(12).

345. Youssef, H.M., et al., *Dissecting the Genetic Basis of Lateral and Central Spikelet Development and Grain Traits in Intermedium-Spike Barley (Hordeum vulgare Convar. Intermedium).* Plants (Basel), 2020. **9**(12).

346. Yao, L., et al., *Combined genome-wide association study and transcriptome analysis reveal candidate genes for resistance to Fusarium ear rot in maize.* J Integr Plant Biol, 2020. **62**(10): p. 1535-1551.

347. Yao, F., et al., *Population structure and genetic basis of the stripe rust resistance of 140 Chinese wheat landraces revealed by a genome-wide association study.* Plant Sci, 2020. **301**: p. 110688.

348. Yang, J., et al., *Unlocking the relationships among population structure, plant architecture, growing season, and environmental adaptation in Henan wheat cultivars.* BMC Plant Biol, 2020. **20**(1): p. 469.

349. Xu, F., et al., *Genome-Wide Association Study on Seminal and Nodal Roots of Wheat Under Different Growth Environments.* Front Plant Sci, 2020. **11**: p. 602399.

350. Wu, D., et al., *Identification of a candidate gene associated with isoflavone content in soybean seeds using genome-wide association and linkage mapping.* Plant J, 2020. **104**(4): p. 950-963.

351. Wang, Y., et al., *Molecular variation in a functionally divergent homolog of FCA regulates flowering time in Arabidopsis thaliana.* Nat Commun, 2020. **11**(1): p. 5830.

352. Wang, X., et al., *Large-fragment insertion activates gene GaFZ (Ga08G0121), and is associated with the fuzz and trichome reduction in cotton (Gossypium arboreum).* Plant Biotechnol J, 2020.

353. Wang, N., et al., *Applications of genotyping-by-sequencing (GBS) in maize genetics and breeding.* Sci Rep, 2020. **10**(1): p. 16308.

354. Van, K., et al., *Mining germplasm panels and phenotypic datasets to identify loci for resistance to Phytophthora sojae in soybean.* Plant Genome, 2020: p. e20063.

355. Valdisser, P., et al., *Genome-Wide Association Studies Detect Multiple QTLs for Productivity in Mesoamerican Diversity Panel of Common Bean Under Drought Stress.* Front Plant Sci, 2020. **11**: p. 574674.

356. Tanaka, N., et al., *Investigation of the genetic diversity of a core collection of japanese rice landraces (JRC) using whole-genome sequencing.* Plant Cell Physiol, 2020.

357. Simeone, R., et al., *Mapping Powdery Mildew (Blumeria graminis f. sp. tritici) Resistance in Wild and Cultivated Tetraploid Wheats.* Int J Mol Sci, 2020. **21**(21).

358. Seck, W., D. Torkamaneh, and F. Belzile, *Comprehensive Genome-Wide Association Analysis Reveals the Genetic Basis of Root System Architecture in Soybean.* Front Plant Sci, 2020. **11**: p. 590740.

359. Satturu, V., et al., *Multiple Genome Wide Association Mapping Models Identify Quantitative Trait Nucleotides for Brown Planthopper (Nilaparvata lugens) Resistance in MAGIC Indica Population of Rice.* Vaccines (Basel), 2020. **8**(4).

360. Ruang-Areerate, P., et al., *Genome-wide association mapping for grain manganese in rice (Oryza sativa L.) using a multi-experiment approach.* Heredity (Edinb), 2020.

361. Ruan, Y., et al., *Characterization of the Genetic Architecture for Fusarium Head Blight Resistance in Durum Wheat: The Complex Association of Resistance, Flowering Time, and Height Genes.* Front Plant Sci, 2020. **11**: p. 592064.

362. Roman-Reyna, V., et al., *Characterization of the Leaf Microbiome from Whole-Genome Sequencing Data of the 3000 Rice Genomes Project.* Rice (N Y), 2020. **13**(1): p. 72.

363. Reddy, V.R.P., et al., *Genome-Wide Association Analysis for Phosphorus Use Efficiency Traits in Mungbean (Vigna radiata L. Wilczek) Using Genotyping by Sequencing Approach.* Front Plant Sci, 2020. **11**: p. 537766.

364. Qu, M., et al., *Alterations in stomatal response to fluctuating light increase biomass and yield of rice under drought conditions.* Plant J, 2020. **104**(5): p. 1334-1347.

365. Plewiński, P., et al., *Innovative transcriptome-based genotyping highlights environmentally responsive genes for phenology, growth and yield in a non-model grain legume.* Plant Cell Environ, 2020. **43**(11): p. 2680-2698.

366. Petit, J., et al., *Genetic Architecture of Flowering Time and Sex Determination in Hemp (Cannabis sativa L.): A Genome-Wide Association Study.* Front Plant Sci, 2020. **11**: p. 569958.

367. Ogbonna, A.C., et al., *Large-scale genome-wide association study, using historical data, identifies conserved genetic architecture of cyanogenic glucoside content in cassava (Manihot esculenta Crantz) root.* Plant J, 2020.

368. Ogawa, D., et al., *Haplotype analysis of data from UAV imagery of rice MAGIC population for the trait dissection of biomass and plant architecture.* J Exp Bot, 2020.

369. Muñoz-Amatriaín, M., et al., *Perspectives on Low Temperature Tolerance and Vernalization Sensitivity in Barley: Prospects for Facultative Growth Habit.* Front Plant Sci, 2020. **11**: p. 585927.

370. Meyer, R.C., et al., *Temporal dynamics of QTL effects on vegetative growth in Arabidopsis thaliana.* J Exp Bot, 2020.

371. Ma, X., et al., *Genetic architecture to cause dynamic change in tiller and panicle numbers revealed by genome-wide association study and transcriptome profile in rice.* Plant J, 2020. **104**(6): p. 1603-1616.

372. Liu, S., et al., *Resequencing of 297 melon accessions reveals the genomic history of improvement and loci related to fruit traits in melon.* Plant Biotechnol J, 2020. **18**(12): p. 2545-2558.

373. Liu, N., et al., *Phenotypic Plasticity Contributes to Maize Adaptation and Heterosis.* Mol Biol Evol, 2020.

374. Liu, J.Y., et al., *An evolutionary population structure model reveals pleiotropic effects of GmPDAT for traits related to seed size and oil content in soybean.* J Exp Bot, 2020. **71**(22): p. 6988-7002.

375. Liu, F., et al., *Haplotype-based genome-wide association increases the predictability of leaf rust (Puccinia triticina) resistance in wheat.* J Exp Bot, 2020. **71**(22): p. 6958-6968.

376. Li, X., et al., *Analysis of genetic architecture and favorable allele usage of agronomic traits in a large collection of Chinese rice accessions.* Sci China Life Sci, 2020. **63**(11): p. 1688-1702.

377. Li, J., et al., *Genome-wide analyses reveal footprints of divergent selection and popping-related traits in CIMMYT's maize inbred lines.* J Exp Bot, 2020. **2020**.

378. Li, B., et al., *Phenomics-based GWAS analysis reveals the genetic architecture for drought resistance in cotton.* Plant Biotechnol J, 2020. **18**(12): p. 2533-2544.

379. Kronenberg, L., et al., *Temperature response of wheat affects final height and the timing of stem elongation under field conditions.* J Exp Bot, 2020.

380. Kolkman, J.M., et al., *Maize Introgression Library Provides Evidence for the Involvement of liguleless1 in Resistance to Northern Leaf Blight.* G3 (Bethesda), 2020. **10**(10): p. 3611-3622.

381. Jin, T., et al., *Natural variation in the promoter of GsERD15B affects salt tolerance in soybean.* Plant Biotechnol J, 2020.

382. Jia, M., et al., *Genome-wide association analysis of stripe rust resistance in modern Chinese wheat.* BMC Plant Biol, 2020. **20**(1): p. 491.

383. Hou, L., et al., *Genome-wide association studies of fruit quality traits in jujube germplasm collections using genotyping-by-sequencing.* Plant Genome, 2020. **13**(3): p. e20036.

384. Gupta, P., et al., *Genomic Regions Associated with the Control of Flowering Time in Durum Wheat.* Plants (Basel), 2020. **9**(12).

385. Guo, Z., et al., *Genetic analyses of lodging resistance and yield provide insights into post-Green-Revolution breeding in rice.* Plant Biotechnol J, 2020.

386. Guo, H., et al., *Differentiation, evolution and utilization of natural alleles for cold adaptability at the reproductive stage in rice.* Plant Biotechnol J, 2020. **18**(12): p. 2491-2503.

387. Griebel, S., A. Adedayo, and M.R. Tuinstra, *Genetic diversity for starch quality and alkali spreading value in sorghum.* Plant Genome, 2020: p. e20067.

388. Geng, X., et al., *Genome-wide dissection of hybridization for fiber quality- and yield-related traits in upland cotton.* Plant J, 2020. **104**(5): p. 1285-1300.

389. Gao, J., et al., *GmCCD4 controls carotenoid content in soybeans.* Plant Biotechnol J, 2020.

390. Fu, L., et al., *Genome-wide association analysis of stem water-soluble carbohydrate content in bread wheat.* Theor Appl Genet, 2020. **133**(10): p. 2897-2914.

391. Fatima, F., et al., *Identification of New Leaf Rust Resistance Loci in Wheat and Wild Relatives by Array-Based SNP Genotyping and Association Genetics.* Front Plant Sci, 2020. **11**: p. 583738.

392. Dossa, K., et al., *A novel motif in the 5'-UTR of an orphan gene 'Big Root Biomass' modulates root biomass in sesame.* Plant Biotechnol J, 2020.

393. Diaz-Garcia, L., et al., *Genotyping-by-Sequencing Identifies Historical Breeding Stages of the Recently Domesticated American Cranberry.* Front Plant Sci, 2020. **11**: p. 607770.

394. Diaz, S., et al., *Genetic mapping for agronomic traits in a MAGIC population of common bean (Phaseolus vulgaris L.) under drought conditions.* BMC Genomics, 2020. **21**(1): p. 799.

395. Dang, X., et al., *OsSYL2(AA) , an allele identified by gene-based association, increases style length in rice (Oryza sativa L.).* Plant J, 2020. **104**(6): p. 1491-1503.

396. Caproni, L., et al., *European landrace diversity for common bean biofortification: a genome-wide association study.* Sci Rep, 2020. **10**(1): p. 19775.

397. Bhattarai, G., et al., *Genome Wide Association Studies in Multiple Spinach Breeding Populations Refine Downy Mildew Race 13 Resistance Genes.* Front Plant Sci, 2020. **11**: p. 563187.

398. Ben Hdech, D., et al., *Exploring natural diversity of Medicago truncatula reveals physiotypes and loci associated with the response of seedling performance to nitrate supply.* Physiol Plant, 2020. **170**(2): p. 227-247.

399. Begum, H., et al., *Genetic dissection of bread wheat diversity and identification of adaptive loci in response to elevated tropospheric ozone.* Plant Cell Environ, 2020. **43**(11): p. 2650-2665.

400. Banoo, A., et al., *North-Western Himalayan Common Beans: Population Structure and Mapping of Quantitative Anthracnose Resistance Through Genome Wide Association Study.* Front Plant Sci, 2020. **11**: p. 571618.

401. An, Y., et al., *Genome-wide association studies and whole-genome prediction reveal the genetic architecture of KRN in maize.* BMC Plant Biol, 2020. **20**(1): p. 490.

402. Almeida, C.P., et al., *Genetic Diversity, Population Structure, and Andean Introgression in Brazilian Common Bean Cultivars after Half a Century of Genetic Breeding.* Genes (Basel), 2020. **11**(11).

403. Adhikari, P., et al., *Genetic variation associated with PPO-inhibiting herbicide tolerance in sorghum.* PLoS One, 2020. **15**(10): p. e0233254.

404. Li, W., et al., *A Natural Allele of a Transcription Factor in Rice Confers Broad-Spectrum Blast Resistance.* Cell, 2017. **170**(1): p. 114-126.e15.

405. Liu, W., et al., *Genome-wide association mapping reveals a rich genetic architecture of stripe rust resistance loci in emmer wheat (Triticum turgidum ssp. dicoccum).* Theor Appl Genet, 2017. **130**(11): p. 2249-2270.

406. Joukhadar, R., et al., *Genome-wide association reveals a complex architecture for rust resistance in 2300 worldwide bread wheat accessions screened under various Australian conditions.* Theor Appl Genet, 2020. **133**(9): p. 2695-2712.

407. Al-Tamimi, N., et al., *Salinity tolerance loci revealed in rice using high-throughput non-invasive phenotyping.* Nat Commun, 2016. **7**: p. 13342.

408. Kuroha, T., et al., *Ethylene-gibberellin signaling underlies adaptation of rice to periodic flooding.* 2018. **361**(6398): p. 181-186.

409. Exposito-Alonso, M., et al., *Natural selection on the Arabidopsis thaliana genome in present and future climates.* Nature, 2019. **573**(7772): p. 126-129.
